# Anelloviruses: From General Biology to Their Role as Biomarkers of Immune Competence in HIV Infection

**DOI:** 10.3390/v18020235

**Published:** 2026-02-13

**Authors:** Alina R. Nokhova, Kirill A. Elfimov, Alexander M. Shestopalov, Natalya M. Gashnikova, Olga G. Kurskaya

**Affiliations:** 1 Federal Research Center of Fundamental and Translational Medicine, Novosibirsk 630060, Russia; 2 State Research Center of Virology and Biotechnology ‘Vector’, Koltsovo 630559, Russia; elfimovkiril@yandex.ru (K.A.E.);

**Keywords:** anelloviruses, HIV, anellome, HIV infection, ART, TTV, immune status

## Abstract

Viruses of the family *Anelloviridae* represent a predominant component of the human virome across various anatomical sites, yet their clinical significance remains poorly understood. This review summarizes current data on the dynamics and functional interactions of anelloviruses with the immune system in the context of human immune deficiency virus (HIV) infection. Existing studies indicate that an individual’s complement of anelloviruses (their “anellome”) serves as a highly sensitive indicator of immunocompetence. In the absence of antiretroviral therapy (ART), the viral load and taxonomic diversity of anelloviruses (genera *Alphatorquevirus*, *Betatorquevirus*, and *Gammatorquevirus*) demonstrate a rapid increase, correlating with HIV viral load, a decline in CD4+ T-lymphocyte count, and the CD4/CD8 ratio, reflecting weakened immune surveillance. Upon initiation of antiretroviral therapy (ART), a decrease in anellovirus viral load is observed; however, it likely does not revert to the pre-HIV infection baseline. At the same time, a high baseline level of Torque teno virus (TTV) is associated with incomplete immune recovery and the risk of ART non-response. Anelloviruses exhibit a dual role as both activators of the immune system (via APOBEC3, antibody production, and pro-inflammatory cytokines resulting from Toll-like receptor (TLR) activation) and disruptors of certain signaling pathways (through micro-RNAs and proteins encoded by ORF2). Thus, monitoring the anellome represents a promising non-invasive approach for assessing immune status, risk stratification, and personalizing therapy in patients with HIV infection. Future research should focus on the practical application of anellovirus viral load and diversity as markers of immune status and on clarifying the consequences of the aggregate interaction between HIV modulator proteins and anelloviruses during co-infection.

## 1. Introduction

**Figure 1:**
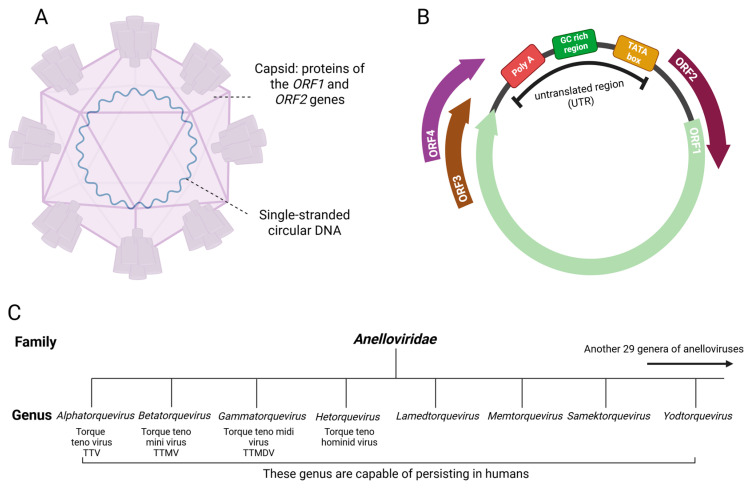
Graphical representation of the general characteristics of anelloviruses: (**A**) schematic structure of the anellovirus virion (icosahedral virion with single-stranded circular DNA); (**B**) schematic representation of the anellovirus genome with the untranslated region (UTR) and open reading frames (ORF1, ORF2, ORF3, putative ORF4); (**C**) classification of anelloviruses that infect humans.

The family *Anelloviridae* comprises a vast group of viruses with single-stranded circular DNA (ssDNA) and a small, non-enveloped icosahedral virion measuring approximately 30 nm in diameter (Figure 1A) [1]. The ssDNA ranges from 2.0 to 3.9 kilobases in size. Genome size is variable and depends on the genus: *Alphatorquevirus* genomes are approximately 3.6–3.9 kb, *Betatorquevirus* 2.8–2.9 kb, and *Gammatorquevirus* 2.0–3.2 kb [2]. Viruses of the family *Anelloviridae* are collectively referred to as anelloviruses. Members of its constituent genera are commonly denoted by their abbreviations (Figure 1C): TTV (*Alphatorquevirus*), TTMV (*Betatorquevirus*), and TTMDV (*Gammatorquevirus*).

The genome contains several open reading frames (ORFs). ORF1 encodes the capsid genes [3]; ORF2 encodes genes involved in immune modulation. The precise functions of the proteins encoded by ORF3 and ORF4 are not fully understood (Figure 1B). The ORF3 product is a serine-rich protein with arginine-lysine-rich domains in its C-terminal region; it contains numerous nuclear localization signals, which is supported by its homology to DNA topoisomerase I [4]. The function and composition of the hypothetical ORF4 product remain unconfirmed, though it is predicted to be a transcriptional regulator localized to the nucleus [5].

Classification of members within the family into genera is based on sequence divergence in ORF1. Recent taxonomy proposes a species demarcation threshold of ~69% nucleotide identity in ORF1 [6]. At the time of publication, ICTV describes 37 genera of *Anelloviridae* and approximately 240 species. Eight genera are found in humans (Figure 1C): *Alphatorquevirus* (Torque teno virus, TTV), *Betatorquevirus* (Torque teno mini virus, TTMV), *Gammatorquevirus* (Torque teno midi virus, TTMDV), *Hetorquevirus* (Torque teno hominid virus), *Lamedtorquevirus*, *Memtorquevirus*, *Samektorquevirus*, and *Yodtorquevirus* [3,7,8].

Viruses of the family *Anelloviridae* persist in the majority of humans. Infection with anelloviruses occurs very early in life: they are detected in infants and are typically acquired within the first few months. After childhood, every adult individual harbors a personal set of anelloviruses (anellome), whose overall composition remains remarkably stable over decades (≈30 years) [9]; this long-term persistence, however, does not exclude short-term fluctuations or gradual replacement of strains, and can be disrupted by significant immunological disturbances (iatrogenic immunosuppression, acquired immunodeficiency syndrome (AIDS), which are discussed below and systemic lupus erythematosus [10,11], rheumatoid arthritis [12]).

A direct pathogenic role for human anelloviruses has not been established. Although the first discovered TTV was isolated from a patient with post-transfusion hepatitis, its exceptionally high prevalence in the healthy general population argues against it being the direct etiological agent of a specific disease. Consequently, these viruses are predominantly considered commensal components of the human virome. Anelloviruses demonstrate the ability for permanent persistence: the immune system possesses multiple factors controlling their numbers, while the viruses themselves possess evasion mechanisms (e.g., miRNA and ORF2/ORF3 proteins) [13]. This phenomenon allows anelloviruses to be considered a potential marker for assessing human immune status.

Immunodeficiency can arise from the use of specific medications following organ or hematopoietic stem cell transplantation, or from an infectious process caused by the human immunodeficiency virus (HIV). Chronic HIV infection leads to severe damage to the immune system in the absence of treatment (AIDS), characterized by massive loss of specific cells, primarily CD4+ T cells [14]. The administration of antiretroviral therapy (ART) and effective suppression of HIV viral load helps to preserve immune status. However, several markers indicate incomplete immune recovery compared to healthy individuals even in this case. For example, HIV-positive individuals exhibit a higher number of exhausted (PD-1+, TIM-1+, CCL5+) cells compared to HIV-negative individuals (the difference is about three times) [15,16,17]. Concurrently, chronic systemic immune activation and elevated production of pro-inflammatory cytokines are observed. There are challenges in assessing the functional state of the immune system that extend beyond common clinical tests, such as measuring CD4+ T-cell count [18], the CD4/CD8 ratio [19], and HIV viral load [20]. Normal values of these parameters do not necessarily indicate complete immune restoration. At the same time, more detailed information about the state of the immune system could help adjust the prescribed ART.

Information on the diversity and viral load of an individual’s personal anellome is therefore being investigated as a potential candidate to fill this gap, as anelloviruses are present in virtually all people, and their anellome remains stable under a normally functioning immune system. While an individual’s pre-infection baseline would be ideal, its utility does not strictly depend on it. Clinically, the anelloviruses (e.g., viral load) could be interpreted like other quantitative biomarkers: by comparing a patient’s current values against established reference ranges for healthy or immunocompetent populations. It is known that infectious processes affect the anellome. For instance, during SARS-CoV-2 infection, the quantity and diversity of anelloviruses decreased several days before symptom onset and continued to decline during the first four weeks after their appearance [21]. Furthermore, certain properties of anelloviruses suggest a modulatory role in immunity. For instance, TTV can suppress the NF-κB signaling pathway and reduce the production of certain inflammatory cytokines by about 4 times (IL-6, IL-8, COX-2) [13,22].

In light of the above, anelloviruses may play a significant role during HIV infection. Firstly, this is due to their importance as markers of the functional state of the immune system, particularly peripheral blood mononuclear cells. Secondly, it is due to their potential to modulate signaling pathways critically involved in pro-inflammatory responses (e.g., IL-6) and HIV-1 transcription processes (e.g., NF-κB). This review aims to systematize and describe the existing data on the human peripheral blood anellome during HIV infection.

## 2. Dynamics of the Human Anellome

### 2.1. Transmission Routes of Anelloviruses

Currently, the parenteral route is considered one of the most likely modes of transmission. This assumption is supported by a number of studies demonstrating a significant increase in viral load following blood transfusion [23,24,25]. An additional argument is the ability of anelloviruses to replicate in bone marrow cells and peripheral blood mononuclear cells (PBMCs) [26], suggesting their blood-borne transmission. The most direct evidence for parenteral transmission comes from a study among people who inject drugs, which revealed similar anellome profiles among individuals within the same needle-sharing cluster. This indicates cross-transmission of the virus through shared needles and confirms the possibility of parenteral infection [27].

The possibility of sexual transmission of anelloviruses has not been conclusively established and is not described in detail in the current literature, though there are reports of TTV DNA being detected in semen [28] and vaginal secretions [29]. It is important to differentiate between detection of viral nucleic acid and proof of relevant transmission. Detection of anellovirus DNA in the genital tract indicates presence of viral genomes, but does not by itself demonstrate replication-competent virus or confirm transmission between individuals via sexual contact, but this route cannot be ruled out completely. Anelloviruses are presumed to have broad tissue tropism [4], with evidence of their ability to persist in hematopoietic stem cells and blood granulocytes [30] and to replicate in stimulated (e.g., by IL-2, phytohemagglutinin, lipopolysaccharides) mononuclear cells, though replication is not observed in non-stimulated cells [31]. This ability to infect immune cells could hint at a potential for sexual transmission, as various immune cells are present in the genital tract mucosa [32]. This mode of spread would be analogous to HIV, which infects CCR5+ T-cells, immune-competent cells located in the mucosal barriers of the genital tract [33].

Vertical (mother-to-child) transmission of anelloviruses remains a subject of debate. On one hand, several studies provide circumstantial evidence supporting the possibility of such transmission. For example, Matsubara et al. report that TTV and TTMV DNA was detected in the cord blood of 48.1% of newborns, with all infected newborns being born to TTV-positive mothers, and the complete homology of viral sequences in mother-child pairs strengthens the case for vertical transfer, although by itself is not definitive proof of in transplacental transmission. A significant argument was also the detection of viral DNA in 100% (6/6) of analyzed amniotic fluid samples in the same study [34]. These data are consistent with the results of other studies: Bagaglio et al. detected TTV DNA in the serum of newborns immediately after birth with 88% sequence homology in mother-infant pairs [35], and Gerner et al. identified TTV in 13.8% of cord blood samples [36]. On the other hand, a separate study conducted in a population with a high frequency of maternal infection did not detect the virus in newborn blood [37]. Thus, although the collective indirect data from various studies indicate the possibility of vertical transmission, demonstrating the detection of viral DNA in cord blood and amniotic fluid with varying frequency, the contradictory results highlight the need for further research. An important factor explaining this discrepancy may be the use of different test systems with varying sensitivity and specificity, as shown in the work by Matsubara and colleagues [34].

An alternative hypothesis of infection during and after childbirth is also discussed. For example, it is known that anelloviruses have been detected in vaginal secretions and cervical samples from healthy pregnant women, confirming the potential for newborn exposure to the virus during vaginal delivery [29,38]. Furthermore, children born vaginally have a higher anellovirus viral load compared to children born via cesarean section [39]. These facts constitute indirect evidence supporting the potential transmission of the virus to the child during passage through the birth canal.

Another proposed mechanism of infection involves the transmission of anelloviruses from mother to child through breastfeeding. One study showed that anellovirus DNA was detected in the breast milk of 67% of lactating mothers [34]. Moreover, TTMV and TTMDV are detected more frequently in breast milk (~32% of samples) and TTV significantly less often (~10%), which correlates with the dominance of *Betatorquevirus* and *Gammatorquevirus* in early childhood [40].

However, reports of a high degree of similarity (up to 50%) between the anellomes of a child and mother following cesarean section and in the absence of breastfeeding suggest the existence of alternative transmission routes during the first months of life [41].

One such potential transmission route could be the fecal–oral pathway. Anelloviruses are detected in the feces of both children [42] and adults [43], indicating the potential for virus transmission via contaminated hands, objects, or food.

The frequent initial detection of anelloviruses in the upper respiratory tract and only subsequently in blood, is consistent with the hypothesis that the respiratory tract may serve as a primary entry site or reservoir, potentially implicating a respiratory droplet transmission route [44]. This hypothesis is further supported by the common detection of anellovirus genetic material (DNA) in saliva [45] and nasal secretions. For instance, our study of children with pneumonia demonstrated such detection [46], as did a study in Shanghai where anelloviruses were found in 63.3% of children with respiratory infections, primarily as a persistent component of the respiratory virome [47]. Importantly, the work of Chikasue et al. [48] detected TTV DNA in exhaled breath condensate and indoor air samples, providing evidence for the presence of viral genomes in aerosols. Airborne (aerosol) transmission is considered one of the probable routes in other studies as well [49,50]. Taken together, these observations make respiratory droplet transmission plausible, especially in settings with close interpersonal contact. However, epidemiologically confirmed evidence of transmission by this route has not yet been obtained.

**Figure 2:**
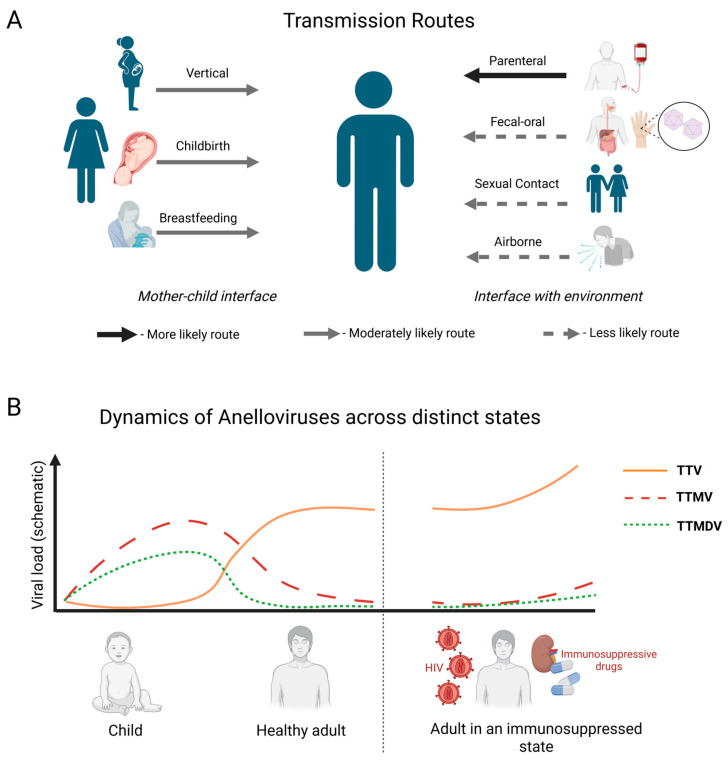
Schematic representation of transmission and viral load dynamics of anelloviruses (*Alphatorquevirus* (TTV), *Betatorquevirus* (TTMV), and *Gammatorquevirus* (TTMDV)) during human ontogenesis: (**A**) Potential routes of transmission of anelloviruses. Arrow style encodes the strength/type of supporting evidence from the literature: solid black—highest confidence (donor → recipient sequence homology and reproducible post-exposure increases in viral load); solid gray—moderate confidence (sequence homology or/and consistent cohort associations and PCR detection in relevant sample types (amniotic fluid, vaginal secretions, etc.)); dashed gray—limited evidence (PCR detection only in relevant sample types). (**B**) Dynamics of viral load of anelloviruses of various genera in three distinct states: early life (child), healthy adult (stable immune status) and adult in immunosuppressed state (examples: HIV infection, immunosuppressive therapy). Curves are illustrative schematic representations based on published tendencies.

Thus, while multiple potential transmission routes for anelloviruses are supported by detection of viral genomes in a variety of tissues and secretions, the strength of evidence varies by route. The presumed mechanisms of spread include parenteral, respiratory droplet, fecal–oral, and vertical transmission, as well as transmission through breast milk (Figure 2A). Some data also point to the possibility of sexual transmission, although this route has been studied much less extensively. Although the question of dominant transmission pathways and the relative contribution of each requires further investigation, the multiplicity of proposed mechanisms may explain the extremely high prevalence of anelloviruses within the human population.

### 2.2. Dynamics of the Composition and Viral Load of the Anellome

*Anelloviridae* is widespread among humans, but the highest viral load in adults is observed for members of the genus TTV. It is known that TTV viral load increases in the setting of immunodeficiency of various origins. For example, this occurs with the use of immunosuppressants after organ transplantation [23]. One study demonstrated that after kidney transplantation, plasma TTV viral load in patients increased from ~4.5 log10 DNA copies/mL to ~8.4 log10 copies/mL [51]. Paradoxically, this increase in viral load can be accompanied by a decrease in TTV species diversity within a single patient’s sample. The median number of *Alphatorquevirus* species fell from 5 to 2. In some patients, a complete shift in the TTV species composition was noted: species circulating before transplantation were not detected post-procedure, while new species, undetected prior to transplantation, were found [51]. This observed shift in diversity and composition of TTV dynamics could be explained by two non-exclusive hypotheses: (I) under reduced immune surveillance, strains with a replicative advantage may undergo selective expansion, outcompeting others and reducing overall diversity; or (II) it might reflect the transmission and establishment of new TTV lineages from the donor.

This paradigm of increased viral load under immunosuppression, however, may not be universal. Importantly, the relationship between anellovirus load and immune status appears to be context-dependent and not unidirectional. While elevated loads are typically observed in long-term immunosuppression (e.g., post-transplant, HIV), some data suggest different patterns may occur in other clinical settings. A longitudinal study in critically ill patients with brain injury, measuring viral loads of TTV, TTMV, TTMDV during the first 2.5 weeks of intensive care, found that the presence of hospital-acquired pneumonia or acute respiratory distress syndrome was associated with relatively lower overall anellovirus DNA levels in blood compared to patients without these complications [52]. This association was not statistically significant for TTV alone in blood, nor was it observed in respiratory tract samples. The authors hypothesize that this reduction could reflect specific immune or inflammatory conditions in early critical illness.

Another study focusing on the impact of smoking and alcohol consumption on TTV viral load in stable kidney transplant recipients (without active infections or malignancies) showed that TTV viral load was lower in patients who were current smokers and had an alcohol intake of >20 g per day [53]. It is established that alcohol consumption [54] and smoking [55] negatively affect the functional state of the immune system. Nevertheless, it was noted that smoking and alcohol consumption of >20 g per day showed a negative correlation with TTV viral load [53], whereas immune dysfunction typically increases TTV viral load. This counterintuitive observation suggests a more complex, context-dependent relationship between immune function and the virome. Thus, future research should investigate whether integrating baseline virome profiles and lifestyle factors could improve the interpretative power of the anelloviruses as a dynamic marker for therapy adjustment.

The composition of the anellome is not uniform throughout the human lifespan (Figure 2B). In early childhood, the anellome is typically dominated by the genera *Betatorquevirus* (TTMV) and *Gammatorquevirus* (TTMDV). However, a characteristic dynamic is observed as the child develops: the viral load of TTV increases rapidly (from 10^4^ copies to 10^5^ copies of TTV DNA/mL) and begins to predominate over TTMV and TTMDV by 3–6 months of age. The establishment of persistent human anellovirus colonization concludes around six months to one year, and by the age of 12–18 months, the anellome is established in almost all infants, reaching a prevalence close to 100% [56]. One study points to the possibility of very early transmission, demonstrating the detection of anelloviruses in the blood of newborns in cases of pregnancy complications such as histological chorioamnionitis, spontaneous preterm birth, and preeclampsia. However, the authors emphasize the preliminary nature of these findings and the need for further investigation into the link between TTV and perinatal pathology [37].

The composition and abundance of anelloviruses are also influenced by other factors, such as sex and age. One study demonstrated that the number of anellovirus variants in men is approximately twice as high as in women. This disparity was particularly pronounced among younger individuals, where the difference in diversity reached threefold. The number of anelloviruses also increased with the age of the subjects: approximately 8 species were detected in 20-year-old men, compared to about 11–12 species in men over 60; around 2 species were found in 20-year-old women, while 60-year-old women also had about 12 species. It is noteworthy that, according to the researchers, the number of detectable anelloviruses in men increased linearly with age, whereas in women, an exponential growth was observed between the ages of 20 and 60 [57].

## 3. Interactions of Anelloviruses with the Immune System

### 3.1. Control of Anelloviruses

**Figure 3:**
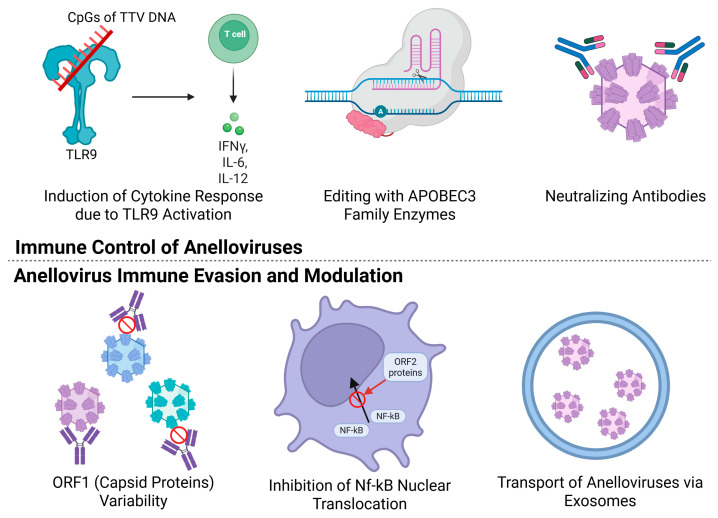
Schematic representation of the main processes involved in the interaction between anelloviruses and the human immune system. These interactions are divided into control of the viral load of anelloviruses by the immune system (activation of TLR9 and production of pro-inflammatory cytokines; editing of anellovirus DNA by APOBEC3 proteins; production of neutralizing antibodies) and evasion of immune control by anelloviruses (variability of ORF1 gene capsid proteins; inhibition of the NF-κB signaling pathway by disrupting its nuclear translocation; transport in peripheral blood as part of exosomal particles [58], in which virions are able to avoid encountering antibodies).

Anelloviruses engage various components of the immune system, involving humoral and cellular, as well as innate and adaptive immunity (Figure 3).

Upon activation of innate immunity, classical pathogen recognition mechanisms are engaged: anelloviruses are capable of activating inflammasomes, which are multicomponent protein complexes of the innate immune system that interact with pathogen-associated molecular patterns (PAMPs). PAMP recognition is carried out by pattern recognition receptors (PRRs), particularly Toll-like receptors, among which TLR-9 holds significant importance. TLR-9 is one of the most crucial Toll-like receptors because it leads to the production and release of a range of pro-inflammatory cytokines (IFN-alpha, IL-6, IL-12). The PAMP target for TLR-9 is the CpG islands in the anellovirus genome, which contain unmethylated DNA motifs not typically found in the cytoplasm of human cells [4]. It has been shown that CpG islands of TTV genotype 4 lead to TLR-9 activation [59]. Interestingly, one study demonstrated that this particular TTV genotype was detected only in patients with pneumonia [44]. Some CpG islands, however, exhibit the opposite, inhibitory effect when interacting with TLR-9. Therefore, it is plausible that the quantity and ratio of these stimulatory and inhibitory CpG islands within an individual’s TTV genomes collectively shape the net immunomodulatory effect of the anellome, potentially tilting it toward a more pro- or anti-inflammatory influence. Furthermore, PRRs play a role in maintaining the balance of cellular subpopulations of immune cells in the blood, particularly Th1, Th2, and Th17 cells [50].

Another component of innate immunity that interacts with anelloviruses is the family of APOBEC3 enzyme proteins, which deaminate cytosine (C) to uracil (U) in anellovirus DNA, leading to an accumulation of mutations in the viral DNA that is packaged into viral particles. Such a virion can enter a cell but cannot establish a productive infection, as transcription from the mutated DNA is not possible. The first evidence of TTV DNA editing by this family of enzymes was noted in 1999, where 1 out of 93 TTV reads was edited by APOBEC [60], and these findings were later confirmed in several studies [13]. The proportion of edited reads varies among individuals carrying anelloviruses; a study on two healthy donors demonstrated that in one subject, the number of edited genovariants was 33.96% (18 out of 53 genovariants), while in the second subject it was 18.18% (2 out of 11 genovariants) [61]. A different study, however, presented significantly lower data, indicating that only about 5% of sequences were edited by APOBEC3 [62]. Furthermore, the aforementioned study highlights several key points: (I) editing predominantly occurs on the minus-strand DNA (relative to the viral genome), indicating a cytoplasmic localization of the process (late stages of virion assembly/release); (II) the most frequent editing motif is 5′-TC-3′; (III) the rate of APOBEC3 editing is not stable: it is observed in bursts rather than continuously, and most edited variants do not become fixed in the population and disappear [61].

Anelloviruses are also susceptible to components of the adaptive immune system. Typically, antibodies are directed against the surface proteins of the anellovirus virion, which are encoded by the ORF1 gene [1]. Recent data indicate that TTV-specific antibodies are commonly detectable, and that antibody reactivity directed to the variable P2 spike region of the capsid is associated with reduced viral richness, suggesting that humoral responses can contribute to partial control of infection [63]. It is known that antibodies can also be produced against ORF2 gene products, though in lesser quantities [64]. However, antibody responses appear to be often delayed, strain-specific and incomplete, so humoral immunity alone generally does not lead to eradication of the anellovirus diversity.

It should be noted that ORF2 products are apparently modulator proteins containing nuclear localization signal patterns at their N-terminus. Likely for this reason, antibodies against ORF2/1 and ORF2/2 proteins may be incapable of controlling anellovirus viral load. Information regarding antibodies against the products of the final gene, ORF3, is absent, and furthermore, no monoclonal antibodies available for purchase exist. This complicates the study of the localization and expression dynamics of these proteins during the viral life cycle.

Anelloviruses can evade the humoral immune response through several mechanisms. First, the vast majority of anellovirus proteins (85%) showed no immunogenicity in any of the 156 individuals examined in one study, and following blood transfusion, none of the transmitted anelloviruses elicited an immune response in 3 out of 5 recipients [64]. There is also an opposing viewpoint, according to which all TTV proteins are immunogenic; this study, however, was conducted using recombinant proteins and serum from adult blood donors [65].

Second, the issue of cellular tropism for anelloviruses remains unresolved. It is plausible that their replication could occur in anatomically and/or immunologically privileged tissue reservoirs, which would be difficult to access and could facilitate immune evasion. For instance, anelloviruses have been detected in male ejaculate [66] and cerebrospinal fluid [67].

Third, anelloviruses can travel in the blood plasma within extracellular vesicles, which protects them from antibodies. The average TTV DNA quantity in vesicles containing it was 4.8 × 10^3^/mL, and plasma samples from patients containing such vesicles also had a higher total TTV viral load: 9.6 × 10^5^ copies/mL versus 1.5 × 10^3^ copies/mL [58].

Fourth, a cryo-electron microscopy study of anellovirus-like particles revealed that the most variable regions of the ORF1 protein are exposed on the surface of the viral particle and act as “immunological decoys,” diverting the antibody response away from conserved fragments of the capsid proteins. However, these results require further verification, as the study investigated virus-like particles formed by ORF1 proteins in transfected cells [1].

### 3.2. Interactions of Anelloviruses with the Immune System: Potential Modulatory Effects

Anelloviruses not only persist under immune pressure but are also associated with observable changes in immune function. This influence is complex and not fully understood, but it can be divided into two broad categories: stimulation and inhibition.

The stimulation of the immune response via PRRs (TLR-9), induction of synthesis and release of pro-inflammatory cytokines, including interferons, and activation of APOBEC3 have already been described above. It can be added that TLR-9, predominantly expressed in antigen-presenting cells (e.g., dendritic cells and macrophages), recognizes unmethylated CpG motifs in microbial and viral DNA. The functional role of this pathway in TTV recognition was demonstrated in a study showing that purified genomic DNA from a specific TTV genogroup could trigger the release of pro-inflammatory cytokines (IFN-α, IL-6, IL-12) in mouse spleen cells, with the effect being specifically mediated via TLR-9. This immunostimulatory activity was shown to depend on the sequence and frequency of CpG motifs within the viral genome. Notably, some epidemiological data point to a correlation between the presence of TTV strains with such stimulatory motifs and more severe clinical outcomes of bronchopneumonia in children [68]. These severe isolates likely possess a higher frequency of stimulatory CpG sequences compared to isolates from patients with mild respiratory infections [59]. Thus, a parsimonious interpretation of these in vitro and correlative clinical data is that differences in anellovirus genomes might contribute to variation in pro-inflammatory signaling, which could, hypothetically, influence disease severity during co-infections. Interestingly, the viral load dynamics of anelloviruses themselves are sensitive to the host’s interferon response. In patients receiving IFN-α therapy for hepatitis C virus infection, a significant reduction in plasma TTV DNA concentration is observed [13].

Experimental and observational studies have described a range of mechanisms associated with anellovirus infection that may influence immune function. These mechanisms encompass epithelial damage, the activity of immunomodulatory proteins (e.g., ORF2), viral replication in host immune cells, interactions with transcription factors, and correlations with altered lymphocyte profiles such as anergy and depletion [59].

A study conducted in a pediatric cohort with persistent or recurrent pneumonia reported a negative correlation between TTV viral load and the ciliary beat frequency of the upper respiratory tract epithelium. Furthermore, the authors established a positive correlation between viral load and the percentage of epithelial areas with mobile basal bodies, which are a marker of epithelial damage [69]. An earlier study by the same research group did not establish a direct association between TTV viral load and ciliated epithelial damage; however, it reported an associative link between TTV and the presence of bronchiectasis. Among patients without this diagnosis, TTV was detected in 45% of cases (5/11), while in the cohort of patients with bronchiectasis, the detection rate was 96% (26/27). Additionally, a positive correlation was found between TTV plasma viral load and the severity of bronchiectasis [70].

The observed tropism of TTV for immune cells, enabling its replication within them, represents a key area of study for understanding its interplay with the host immune system. The most compelling data concern granulocytes. For example, after allogeneic hematopoietic stem cell transplantation, TTV DNA with high viral load (averaging 29.82 copies/cell) was detected in peripheral blood and bone marrow granulocytes of 100% of recipients. In contrast, TTV was detected in only 33% of cases in the control group. Notably, in recipients, TTV levels increased post-transplantation, peaking around day 100. Based on the observed temporal association, the authors propose a hypothesis that TTV replication is dependent on the recovery of the granulocyte pool, noting that the increase in viral load occurred approximately two weeks after engraftment [25]. The question of TTV tropism for mononuclear cells remains debated. While the study by Kosulin et al. (2018) did not detect TTV in the CD4+ and CD8+ T-lymphocytes, NK cells, or monocytes of healthy individuals [25], other studies report contrasting results. Takahashi et al. (2002) confirmed the presence of TTV DNA in peripheral blood mononuclear cells and granulocytes, and also detected TTV mRNA in neutrophils, indicating active viral transcription [71]. Another study also found TTV DNA in peripheral blood mononuclear cells, not only in the examined HIV-positive patients but also in healthy individuals [72]. Furthermore, the possibility of TTV replication in immune system cells is supported by work where the virus was temporarily cultured in vitro (for about 4 weeks) in activated peripheral blood mononuclear cells and the B-lymphoblastoid Raji cell line [73].

The most clinically relevant finding is the identified positive correlation between TTV DNA levels and the number of senescent T-cells (CD57+), suggesting one plausible hypothesis that TTV replication in peripheral blood mononuclear cells contributes to this cellular senescence [72]. Another report suggests a possible link between anelloviruses and cancer. There is a positive correlation between the levels of TTV and TTMV and the number of NKG2A+ NK-cells, NKG2A+ CD4+ T-cells, and NKG2A+ CD8+ T-cells. The authors note that anelloviruses can have both negative effects (due to cell depletion, activation of TLR9, and the creation of TTMV-RARA chimeric proteins) and positive effects on humans (inducing apoptosis in tumor cells through the TAIP protein and suppressing the NF-κB signaling pathway through the ORF2 proteins) [74]. However, such conclusions should be interpreted with caution, as both the increase in TTV DNA load and the rise in senescent cell numbers could be consequences of HIV infection and cancer itself, meaning these parameters may not be directly causally linked.

Another hypothetical mechanism by which anelloviruses might be associated with immune changes involves the regulatory proteins encoded by ORF2, which have demonstrated immunomodulatory properties in experimental models. Overexpression studies have shown that the ORF2 product can suppress pro-inflammatory NF-κB signaling elicited by TNF-α in several human cell lines (HeLa, HepG2) and in a mouse macrophage line (RAW264.7), acting in a dose-dependent manner. The ORF2 product interacts with the catalytic subunits of kinases—IKKβ and IKKα—which are involved in activating both the canonical and alternative NF-κB pathways. In the canonical pathway, this leads to the retention of p65/p50 in the cytoplasm, while in the alternative pathway, it reduces the formation of p52. These effects result in the suppression of NF-κB-mediated transcription of pro-inflammatory genes, thereby reducing the production of pro-inflammatory cytokines (IL-6, IL-8, COX-2) at the gene transcription stage [22]. Importantly, these observations derive from in vitro overexpression and have not been confirmed in vivo. Therefore, the role of ORF2 in NF-κB suppression should be described as a plausible, but as yet unconfirmed, hypothesis that requires validation in physiologically relevant models.

Findings indicate that ORF2 may have a dual function: alongside immunomodulation, it appears to be essential for viral particle assembly. Therefore, a plausible interpretation of the knockout data is that ORF2 contributes crucial, non-capsid components to the morphogenesis machinery. This implies a significant, and perhaps equally important, role for ORF2 alongside ORF1, encompassing modulatory functions that span from intracellular assembly (e.g., transport, packaging) to extracellular immune modulation [75].

Furthermore, it is known that one of the ORF3 proteins acts as an apoptosis inducer (TTV-derived apoptosis-inducing protein, TAIP). TAIP consists of 105–108 amino acids (for some genotypes, a shortened N-TAIP peptide of 50–59 amino acids are prevalent, for which apoptotic activity has not been definitively established) and is characterized by a high content of proline, threonine, and leucine, with an N-terminus containing a hydrophobic domain. M.H. de Smit and M.H.M. Noteborn suggest that TAIP is structurally similar to apoptin from chicken anemia virus (CAV), providing grounds to expect a similar mechanism of action, although direct evidence is still lacking. Apoptin can form multimers (30–40 monomers) capable of binding DNA and participating in the induction of p53-independent apoptosis in tumor cells. While TAIP demonstrates a similarly p53-independent apoptotic effect in cell culture and induces characteristic apoptotic morphology, it predominantly localizes to the cytoplasm, contrasting with the typical nuclear accumulation of apoptin in transformed cells [76]. These observations to date are largely limited to in vitro systems, and the precise mechanism and in vivo relevance of TAIP remain to be clarified.

Thus, the interaction between anelloviruses and the host immune system is a complex and dynamic process. This includes both immune-mediated control mechanisms (such as TLR-9 recognition, APOBEC3 editing, and antibody responses) and multiple immune evasion strategies employed by the viruses (e.g., high ORF1 variability, low immunogenicity, vesicle transport, and NF-κB pathway suppression via the ORF2 product). Anelloviruses are significant modulators of immune status due to their dual nature: they can both stimulate and suppress pro-inflammatory reactions, as well as potentially damage tissues and contribute to immune dysfunction. The current mechanistic understanding of these processes, however, remains predominantly hypothetical, built on in vitro findings. Notably, clinical observations—such as the correlation between anellovirus load and immune status—lend plausibility to these models and suggest a potential link to human pathophysiology. These correlations transform the in vitro hypotheses into clinically relevant questions, pinpointing the necessity for future research to bridge the gap between cellular mechanisms and in vivo pathogenesis through targeted longitudinal studies.

## 4. Dynamics of the Anellome During HIV Infection

### 4.1. Principles of HIV-1 Infection Pathogenesis

The HIV virion is 100–120 nm in diameter and consists of a host cell-derived lipid bilayer envelope studded with viral glycoproteins (gp120/gp41). Inside, the conical capsid core contains two copies of the positive-sense single-stranded RNA genome and the essential viral enzymes (reverse transcriptase, integrase, and protease). The primary targets for HIV are CD4+ T-cells, which carry the CD4 receptor and a co-receptor—either C-C chemokine receptor type 5 (CCR5) or C-X-C chemokine receptor type 4 (CXCR4)—on their surface [77].

HIV can also infect other cells through the formation of virological synapses, i.e., direct cell-to-cell virus transmission [78], or via exosomes filled with proteins (p24, gp120, reverse transcriptase) and HIV RNA [79]. Infection mediated by free viral particles begins with the interaction of the viral envelope protein gp120 with the CD4 receptor and one of the co-receptors, either CCR5 or CXCR4.

There are two major genetic lineages of the virus, HIV-1 and HIV-2, which crossed over to humans from different primates (chimpanzees and sooty mangabeys, respectively) [80]. HIV-1 is more virulent, causes a more severe form of HIV infection, and has spread worldwide; HIV-2 causes a milder form of the disease, is less transmissible between humans, and is primarily confined to West Africa [81].

Key processes in HIV infection, from a clinical perspective, are characterized by progressive loss of CD4+ T-lymphocytes, chronic immune inflammation, and immune system dysfunction [82]. The primary mechanism of CD4+ T-cell depletion is their direct infection and lysis during viral particle production, as well as chronic immune activation leading to cell death via apoptosis and pyroptosis. A crucial role in sustaining chronic inflammation is played by the translocation of microbial products from the gut lumen into the systemic circulation, caused by early and severe damage to CD4+ T-cells in the gut-associated lymphoid tissue (GALT) [83]. This damage to the intestinal mucosa leads to constant immune stimulation by bacterial products, such as lipopolysaccharides, which contributes to systemic inflammation even under effective antiretroviral therapy. Furthermore, HIV itself and its proteins (such as gp120, Tat, and Nef) can directly activate immune cells and pro-inflammatory signaling pathways, for example, NF-κB [84], thereby enhancing the production of pro-inflammatory cytokines (IL-6, C-reactive protein, and others). Over time, these processes lead to the accumulation of senescent cells (CD28-/CD57+/KLRG1+) [85], an increased risk of neurodegenerative diseases due to the development of HIV-associated neurocognitive disorders, and a decline in the number of CD4+ T-lymphocytes. The terminal stage of HIV infection is AIDS, characterized by opportunistic infections and cancers [86,87].

ART is used to treat HIV infection and preserve immune function. At the molecular level, ART specifically targets key stages of the HIV life cycle: entry, reverse transcription, integration, and enzymatic proteolysis of viral proteins [88]. In the vast majority of cases, combination therapy is employed, which includes two nucleoside reverse transcriptase inhibitors and one drug from another class (e.g., an integrase inhibitor), ensuring effective suppression of HIV replication and, consequently, a reduction in plasma viral load to an undetectable level (less than 20–50 copies/mL) [89]. Suppression of HIV replication and viremia prevents the loss of CD4+ T-lymphocytes. Systemically, the reduction in viral load and the attenuation of inflammation due to antiretroviral therapy lead to improved clinical status, including an increase in CD4+ T-cell count.

There is a significant gap in research concerning the effects of ART drugs on the composition of the human virome, including the peripheral blood virome. Individual reports note that ART for HIV infection leads to a reduction in anellovirus diversity (after an initial increase prior to starting ART); pegiviruses, in contrast, increase in abundance with longer ART duration. ART did not reduce the abundance of a range of viral families (*Parvoviridae*, *Circoviridae*, *Polyomaviridae*, *Genomoviridae*, *Herpesviridae*, *Adenoviridae*) following their initial expansion during the acute and chronic stages of SIV (Simian immunodeficiency virus) infection before treatment [90]. Another study demonstrates that patients on ART do not experience a significant change in anelloviruses diversity. However, an increase in the viral load of Epstein–Barr virus and some bacteriophages (*Staphylococcus phage vB_Clo6*, *Staphylococcus phage IME-SA4*, *Propionibacterium phage Enoki*) was shown. The latter fact may indicate increased intestinal permeability and the translocation of the host bacteria of these phages (e.g., *Staphylococcus aureus*, *Cutibacterium acnes*) into the bloodstream [91].

Unfortunately, it is impossible to draw a definitive conclusion about the direct influence of ART itself on the virome composition, as all these studies were conducted in the context of HIV infection, which also exerts its own effect. To investigate the effect of ART drugs in more detail, it may be necessary to study the human virome in the context of, for example, the prophylactic use of antiretroviral drugs. Such a study would be possible by enrolling participants from key populations, who often take certain ART medications as pre-exposure prophylaxis.

### 4.2. Dynamics of the Anellome in Treatment-Naïve Patients with HIV

**Figure 4:**
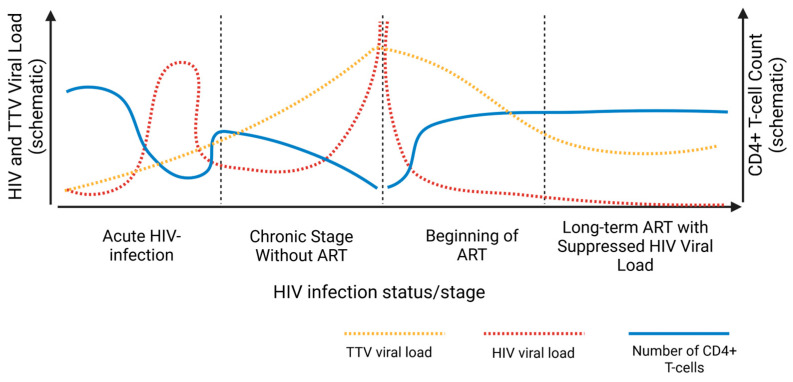
Schematic representation of the key patterns in the dynamics of HIV viral load, TTV viral load, and CD4+ T-cell count at different stages of HIV infection and in the absence/presence of ART. Curves are illustrative summaries of tendencies reported in the literature and do not represent empirical measurements.

During the acute stage of HIV infection (stages Fiebig I-V), a series of processes occur that determine the subsequent course of the disease. Following transmission, the HIV rapidly replicates in mucosal tissues, leading within days to its systemic dissemination and the peak of plasma viremia, where HIV viral load can exceed >10^7^ RNA copies/mL (Figure 4) [92]. This period is characterized by massive depletion of CD4+ T-lymphocytes (Figure 4), particularly within the GALT, which inflicts lasting damage to the immune system. The immune system mounts a strong but often dysfunctional antiviral response, involving the activation of interferon-stimulated genes and the release of numerous pro-inflammatory cytokines and chemokines, such as IFN-α, IL-6, CXCL10, IL-8, and IL-10. Notably, the intensity of this initial inflammatory response correlates with a higher peak HIV viral load and may promote chronic immune activation rather than effective control of viremia [93]. Furthermore, during the acute disease stage, the pool of HIV proviral reservoirs is established, including its latent component, primarily within resting CD4+ T-cells. It is precisely these latent proviral reservoirs that represent the main obstacle to curing HIV [94].

The loss of CD4+ T-cells and massive immune activation are strongly associated with a surge in the viral load and diversity of anelloviruses, particularly TTV (genus *Alphatorquevirus*), as evidenced by the negative correlation between CD4+ count and TTV load [90]. Two potential reasons for this phenomenon can be highlighted: (I) reduced immune surveillance due to the loss of CD4+ T-cells [4]; (II) the superior replicative capacity of TTV in activated peripheral blood mononuclear cells compared to a similar population of non-activated cells [31]. Notably, a parallel expansion of anelloviruses in the intestinal virome has been documented in the context of SIV infection in non-human primates [95], supporting the general link between immunodeficiency and anellome dysregulation.

During acute and chronic HIV infection in the absence of treatment, the detection rate of TTV reaches 100%, and the viral load of anelloviruses is elevated [96]. Furthermore, the TTV viral load shows a positive correlation with HIV viral load and a negative correlation with the CD4+ T-cell count [90,97]. Interestingly, TTV viral load also increases during HIV viremic ‘blip’ (a brief increase in HIV viral load in a patient with previously suppressed viremia, followed by a return to an undetectable level without any change to the ART regimen) [98]. It is important to note that the aforementioned correlations between TTV titers, HIV viral load, and CD4+ T-cell count are observed only in treatment-naïve HIV-positive patients. In patients with suppressed viral load during chronic infection on effective ART, these correlations are lost [99].

A comprehensive analysis of the anellome should consider both the viral load (titer) and the genetic diversity of anelloviruses, as their joint assessment provides a more complete picture of immune system status. Research results indicate that anelloviruses are detected in the vast majority of individuals with HIV infection. One study found that all examined HIV-positive patients (24/24) were infected with TTV and TTMV, and 71% had mixed infections (2–5 genogroups of TTV) [100]. This finding aligns with recent data where TTV detectability also reached 100% (57/57) in HIV-infected individuals [98]. A larger study on a Nigerian cohort (130 HIV-positive and 130 healthy donors) confirmed a statistically significantly higher prevalence of TTV among HIV-positive individuals (65% vs. 26%, *p* < 0.05). Concurrently, TTMV and TTMDV infections were frequent in both groups (≈88–95%), and triple infections (TTV+TTMV+TTMDV) occurred in 60% of HIV-positive subjects versus 22% of healthy donors (*p* < 0.05). The authors also noted high genetic heterogeneity among the Nigerian isolates (≈59% whole-genome identity), underscoring the broad intra-population variability of anelloviruses [101]. The variation in prevalence estimates may be related to differences in detection methods.

Metagenomic analysis of plasma from treatment-naïve HIV-positive individuals also revealed high diversity within the anelloviruses community. The anellome was predominantly represented by the genus *Alphatorquevirus*, with a smaller proportion belonging to the genus *Betatorquevirus* [102]. The generalizability of these findings may be limited, given that the study cohort consisted only of men who have sex with men.

Furthermore, it was observed that the number of CD4+ T cells had a negative correlation with the diversity of anelloviruses. For example, in the group of HIV-positive patients with a CD4+ T-cell count <350 cells/µL, only *Alphatorquevirus* was detected, and representatives of *Betatorquevirus* were found in only one patient from this group. Representatives of the genus *Gammatorquevirus* began to be detected only after the CD4+ T-cell count fell below 350 cells/µL. With a further decline in CD4+ T-cells to <50 cells/µL, anelloviruses of the genera *Alphatorquevirus* and *Betatorquevirus* were detected in almost all samples, alongside a markedly increased frequency of *Gammatorquevirus* detection [103].

Another interesting study was conducted in a group of women living with HIV discordant shedding, where HIV RNA was detected in the cervical canal and vagina despite suppressed plasma HIV viral load. The study detected anelloviruses from three genera as previous manuscript (*Alphatorquevirus*, *Betatorquevirus*, and *Gammatorquevirus*). Notably, the viral load and diversity of anelloviruses were higher in the discordant group compared to both the study’s control cohort and HIV-positive women in whom HIV RNA was not detected in the cervical canal and vagina [104].

Beyond HIV viral load and CD4+ T-cell count, TTV levels have also shown correlation with other immunological markers in people living with HIV. For example, one study found that higher TTV titers were associated with an increased percentage of exhausted B-cells (CD10^−^CD21lowCD27^−^) [105]. The authors suggest this association might mirror a broader impairment in immune surveillance, similar to what is observed with declining CD4+ T-cell counts. It is crucial to note that this observed link is correlational and does not imply causation; elevated TTV load is best interpreted as a robust correlative marker of the underlying immune dysfunction rather than its driver. Therefore, while TTV titer alone does not explain the mechanism of B-cell exhaustion, it serves as a practical, non-causal indicator that may help quantify the overall degree of immune dysregulation [105].

The dynamics of anellovirus viremia are also closely linked to the stage of HIV infection. Studies show that their concentration increases soon after infection. One study demonstrated that during the hyperacute phase, the concentration of TTV DNA in blood mononuclear cells falls between the levels observed in healthy donors and chronic HIV patients (higher than in healthy individuals but lower than in advanced cases) [72]. This indicates that signs of impaired immune surveillance and the associated spread of anelloviruses appear very early, likely even within the HIV seroconversion window and often before a marked decline in CD4+ cell counts.

Anelloviruses rapidly respond to changes in immune status; therefore, it is reasonable to assume that their viral load and diversity possess prognostic potential for assessing treatment efficacy and immune recovery. This was demonstrated, for example, in a study of 301 patients where TTV titers were measured before and after ART initiation. The HIV-positive patients were categorized into several groups based on their remaining CD4+ T-cell count (<50, 50–200, and >200 CD4+ cells/µL). Prior to ART, TTV was detected in 96% of patients. Following treatment, TTV viral load declined, but with varying dynamics in each group stratified by CD4+ T-cell count. It was shown that patients in the group with <50 CD4+ T-cells experienced slower immune recovery compared to the other groups, which was accompanied by a higher TTV viral load [106]. These patients are considered immunological non-responders to ART, characterized by sustained virological suppression (HIV RNA < 50 copies/mL) concurrent with suboptimal immune reconstitution—specifically, a failure to achieve a CD4+ count >500 cells/μL or a CD4+ gain of <200 cells/μL after 24 months of therapy. Immunological non-response is associated with a higher risk of morbidity and mortality [107].

Particular attention should be given to the results obtained from studies of HIV controllers (nonprogressors). This group of patients capable of spontaneously suppressing viral load without ART. One study involving pediatric nonprogressors showed that their level of anellovirus viremia was higher than in patients with a classic progression of HIV infection [108]. This finding contrasts with data from adult cohorts, where the association was reversed: higher anellovirus levels (both in titer and diversity) were observed in cases with a worse clinical presentation. This discrepancy may be related to the fact that a child’s immune system responds to HIV infection somewhat differently. Furthermore, the species composition of anelloviruses was unusual: in contrast to the typical pediatric profile dominated by the genera *Betatorquevirus* and *Gammatorquevirus*, the overwhelming majority of sequences in this cohort belonged to the genus *Alphatorquevirus* [108]. Taken together, these observations support an interpretation wherein the anellome’s association with immune status is not merely a passive marker of immunosuppression but may reflect a more intricate and bidirectional interaction. Finally, this same group of controllers also exhibited elevated overall viral diversity.

Therefore, based on the observations summarized above, anelloviruses (notably TTV) may be considered highly sensitive surrogate markers for tracking immune status throughout untreated HIV infection. Key observations include: (I) The dynamics of viral load and diversity of anelloviruses are early and rapid. Anellovirus expansion begins at the very onset of HIV infection, even prior to seroconversion and a significant decline in CD4+ cell counts. This indicates the initiation of immune surveillance dysfunction.; (II) Clear correlations are observed in adults between anellovirus levels/diversity and markers of HIV progression. These include associations between an expanded, diverse anellome and higher HIV viremia, lower CD4+ counts, and increased numbers of exhausted B-cells. The available data not only confirm the high prevalence of anelloviruses during HIV-induced immunodeficiency but also demonstrate their significant genetic diversity. Consequently, investigating the link between anelloviruses and immune status should consider not just a binary PCR result but also quantitative (viral load) and qualitative (community composition) parameters of the anellome.

Particular interest lies in the seeming paradox identified in the cohort of pediatric long-term non-progressors, where enrichment with anelloviruses (predominantly *Alphatorquevirus*) is associated with a favorable outcome. This contrast with the classical negative correlation underscores that the interpretation of anelloviruses role remains ambiguous and warrants further investigation.

### 4.3. Dynamics of the Anellome During Antiretroviral Therapy (ART)

Despite effective suppression of viral load by ART, complete immune system recovery in HIV-positive patients is typically not achieved. A significant proportion of patients continue to exhibit various immunological abnormalities. Even with undetectable viremia, many patients display a phenomenon of pathological T-lymphocyte proliferation, characterized by excessive cell division where not all progeny are functional immunocompetent cells [109]. Additionally, people living with HIV often demonstrate chronic immune activation and inflammation, as indicated by biomarkers such as the soluble form of the CD163 protein (sCD163), which is generated under the action of TNF-α converting enzyme [110]. sCD163 is associated with an increased risk of non-AIDS-defining illnesses (e.g., type 2 diabetes, cardiovascular disease) [111,112].

A particular challenge is presented by immunological non-responders—patients who fail to restore their CD4+ T-lymphocyte count to levels seen in healthy individuals after ART. This condition is generally associated with late treatment initiation and a low baseline CD4+ T-cell count (<200 cells/μL); its prevalence can reach 50% among patients who started therapy at advanced disease stages [113].

Identified factors for a poor immunological response to ART include older age, male sex, Hepatitis B virus or Hepatitis C virus co-infection, a low baseline CD4+ T-cell count (<200 cells/μL), and a low baseline CD4/CD8 ratio (<0.2) [114]. However, the precise molecular-genetic mechanisms and individual factors leading to this state remain unknown. The human virome, particularly its most abundant component (anelloviruses) may be involved in this process.

The initiation of ART marks a turning point in the dynamics of anelloviruses in HIV-positive patients. Against the backdrop of effective suppression of HIV replication and the beginning of CD4+ T-lymphocyte pool recovery, a corresponding decrease in the plasma viral load of anelloviruses, particularly TTV, is observed. This process reflects a partial restoration of the immune surveillance that normally limits their replication. However, restoring immune surveillance takes time and often does not lead to complete normalization of anellovirus viral load. Even after at least one year of ART, TTV levels typically do not return to their original baseline (i.e., pre-HIV infection levels) [72]. Interestingly, no association was found between the baseline TTV viral load at diagnosis and subsequent CD4+ T-cell recovery, which contradicts the findings of the study by Schmidt et al. discussed earlier [106]. Those authors demonstrated that a high baseline TTV viral load and a lower CD4+ T-cell count at ART initiation had a positive correlation with poorer subsequent CD4+ T-cell recovery.

Another longitudinal study, which monitored HIV-positive patients for 24 months after ART initiation, demonstrated a gradual but significant decrease in the presence of *Anelloviridae* sequences across various biological samples, including plasma and feces. The authors also investigated the dynamics of the patients anellovirus genetic diversity during this period. It should be noted that patients harbored both short-lived and long-lived TTV lineages. Based on data on the duration of TTV lineage circulation during ART, they were categorized into several types: (I) lineages that appear once and persist until the end of observation; (II) lineages that appear once, disappear, but may reappear; (III) lineages that appear once and are subsequently lost for the remainder of the observation period. Another interesting observation concerned the weak positive selection on some lineages, indicated by a greater number of non-synonymous substitutions compared to synonymous ones (dN/dS) in ORF1. The authors concluded that across the entire ORF1 gene, the dN/dS ratio was only slightly above 1 for four out of the six lineages, and only in one TTV lineage did the dN/dS ratio reach 3.5. This suggests that the entire ORF1 gene is under only mild diversifying selection pressure or is evolving neutrally [62]. Notably, anelloviruses are rarely eliminated from the body even after ART achieves an undetectable HIV viral load. Moreover, they persist as one of the most abundant components of the plasma virome [91], saliva [115], and other anatomical reservoirs [103].

Metagenomic analysis of plasma from patients on ART reveals the concurrent circulation of representatives from all three genera (*Alphatorquevirus*, *Betatorquevirus*, *Gammatorquevirus*), as well as multiple phylogenetic groups within each genus. In some individuals, up to eight distinct genetic variants may persist. This indicates a high frequency of co-infections and demonstrates the ability of anelloviruses to evade immune responses, even when the immune system has been partially restored after achieving an undetectable HIV viral load [90].

Nevertheless, more complex changes in the structure of the anellome during ART should also be considered. Despite an overall reduction in the abundance and diversity of anelloviruses, a portion of the population demonstrates high stability. In one study investigating the plasma virome, 654 core lineages of anelloviruses were identified that persisted in the plasma over a 15-month observation period on ART [90]. These anelloviruses were categorized into a personal persistent anellome (lineages detected in ≥75% of a patient’s sampling time points) and a transient anellome (lineages detected in <75% of time points). The anellome was further divided into unique lineages (found in a single patient) and shared lineages (found in 2 or more patients). Moreover, these persistent anelloviruses do not merely survive but continue to evolve. The five most prevalent core lineages accumulated substitutions in the ORF1 gene, evidencing ongoing evolutionary pressure, likely from the partially recovering immune system. Thus, even with successful HIV suppression, key anellovirus populations remain active and continue to adapt [90].

While the anellome underwent transformation, changes in other viral families were less dramatic yet still notable. The virome shift during ART was also characterized by an increased representation of *Flaviviridae*, partly attributable to the expansion of Human Pegivirus 1 (HPgV-1). The expansion of HPgV-1 during ART could be explained by several non-exclusive hypotheses, such as reduced competition for cellular resources from a declining anellome, or the reactivation or reinfection of the virus facilitated by the changing immune landscape. The identified immunological correlations: positive for *Anelloviridae*/*Flaviviridae* with IL-10, IL-2, IL-12, and GM-CSF, and negative with CD4+ cell counts indicate a close link between virome composition and the extent of immune recovery. Notably, most cytokine profile abnormalities did not normalize within the first year of ART. Overall, a one-year course of ART reduces the number and total load of anelloviruses but does not ensure full restitution of a normal plasma virome ecosystem: personalized core anellovirus lineages persist and continue to evolve, and the anellome composition becomes more individualized. Thus, the positive correlations of *Anelloviridae* with certain anti-inflammatory and Th1 cytokines and its negative correlation with CD4+ cell counts [90] support the concept that anellovirus abundance and diversity reflect the degree of “incomplete” immune recovery and may serve as biomarkers of immune status during ART.

A logical extension of this idea is the predictive potential of anelloviruses. Specifically, the prognostic value of the baseline anellovirus viral load prior to therapy initiation is an important and potentially significant clinical aspect. Studies have shown that high baseline TTV viral load and *Anelloviridae* diversity are predictors of suboptimal immunological recovery, correlating with CD4+ T-cell counts, the CD4/CD8 ratio, concentrations of pro-inflammatory cytokines, and the number of senescent cells. The interpretation is conceptually straightforward and empirically supported: the higher the pre-treatment TTV viral load, the poorer the recovery of immune functions during highly effective ART. This manifests as slower CD4+ T-cell count increases, a lower CD4/CD8 ratio, and a higher risk of falling into the category of immunological non-responders who fail to fully restore CD4+ T-lymphocyte counts to a level of ≥350 cells/μL [105,115].

However, the translation of this predictive potential into clinical practice requires careful consideration of conflicting evidence and its sources. For instance, while some researchers identified a high baseline TTV load as a predictor of poor CD4+ T-cell recovery [106], other researchers found no such association [72]. These discrepancies likely stem from methodological and clinical heterogeneity across studies. Methodologically, the quantification of TTV lacks standardization, with different qPCR assays targeting diverse genomic regions yielding variable viral load estimates [116]. Furthermore, the high genetic diversity of TTV nucleotide sequences poses a significant challenge for designing qPCR assays capable of detecting all known genotypes within this and other anellovirus families. Clinically, cohorts differ in key aspects such as the definition of immunological non-response, the timing of ART initiation (early vs. late presenters), baseline CD4+ strata, and the prevalence of co-infections—all established confounders for immune recovery [113,114]. Furthermore, differences in ART regimens and adherence, though less studied in this context, may also influence reconstitution kinetics [88,89]. Therefore, the prognostic signal of baseline TTV is context dependent.

When using the anelloviruses as a marker of immune reconstitution in HIV infection, the choice of biological specimen must be considered, as the quantity and diversity of viruses comprising the human anellome differ across anatomical reservoirs. Research indicates that anelloviruses can persist in other body tissues and fluids. For example, analysis of saliva from patients on long-term ART continues to detect TTV, and its titer maintained an inverse correlation with CD4+ T-lymphocyte levels [115], whereas the correlation between plasma TTV load and CD4+ count typically loses statistical significance with ART [117]. This suggests the potential utility of monitoring salivary TTV viral load as a non-invasive method for assessing immune status in patients with suppressed HIV viremia. This approach is simpler than measuring exhausted cell counts [118] or quantifying HIV proviral reservoirs [119].

The disappearance of statistically significant correlations between TTV viral load and both CD4+ T-cell count and HIV viral load is viewed as indicative of restored immune control necessary for returning the anellome to pre-infection baseline levels or values close to them. Notably, in patients with long-term virological suppression, no significant association is found between the relative abundance of anelloviruses in plasma and the level of T-cell activation (HLA-DR+CD38+) [117]. This suggests that the mere persistence of anelloviruses is not the primary driver of chronic immune inflammation in this patient category, and the leading role in sustaining immune activation likely belongs to other factors, such as microbial translocation from the gut or persistent viral reservoirs.

## 5. Discussion

The presented data review suggests that anelloviruses should not be regarded merely as a passive component of the human virome, but rather as a dynamic and highly sensitive system reflecting the functional state of immunity [13]. In the context of HIV infection, the anellome has been proposed as a unique and informative marker that is both measurable and informative [106]. Key evidence for this is the rapid expansion of anelloviruses, particularly TTV, during the earliest stages of HIV infection, often preceding a significant decline in CD4+ T-lymphocyte counts [72]. This dynamic indicates that the impairment of immune surveillance, which allows anelloviruses to replicate unchecked, is one of the initial consequences of HIV infection [4].

The complex and dual interaction of anelloviruses with the host immune system adds another layer to their potential influence on HIV pathogenesis. On one hand, anelloviruses are under strict immune control, evidenced by their sensitivity to interferon action [13], genome editing by APOBEC3 proteins [61], and antibodies [64]. On the other hand, they have evolved numerous evasion mechanisms, including high variability of the ORF1-encoded capsid proteins [1], low immunogenicity of most of their proteins [64], and the ability to travel within extracellular vesicles in the bloodstream [58]. Furthermore, their modulator proteins, such as those encoded by ORF2, have been shown to suppress key pro-inflammatory pathways in experimental models, particularly NF-κB pathway [22]. Given that this same pathway is activated by HIV modulator proteins (e.g., Tat) to enhance its own replication [84], a hypothesis emerges regarding potential competitive interplay between the immunomodulatory mechanisms of HIV and anelloviruses, which could exert either a negative or positive influence on the course of the primary infection. Beyond this interplay at the level of signaling pathways, an intersection may exist via another arm of the intrinsic immune defense—the APOBEC3 system.

Building on the role of APOBEC3, an intriguing intersection with HIV biology emerges. While anelloviruses bear APOBEC3-associated DNA editing signatures, HIV encodes the Vif (viral infectivity factor) protein, which antagonizes several APOBEC3 family members by promoting their ubiquitination and proteasomal degradation [120]. Given that anellovirus loads generally increase during untreated HIV infection, one possible, though not exclusive, contributing factor could be that Vif-mediated attenuation of APOBEC3 activity could reduce editing of anellovirus genomes, thereby permitting higher anellovirus replication. At present, the net impact of HIV infection on APOBEC3 editing of anelloviruses, and the downstream consequences, remains unresolved and represents a compelling avenue for further study.

The dynamics of the anellome undergo significant changes during ART. Effective suppression of HIV replication and the initiated restoration of the immune system lead to a corresponding decrease in TTV viral load [72]. However, this decrease is often incomplete. A high baseline TTV titer before ART initiation serves as a predictive marker for suboptimal immunological recovery, being associated with a slower increase in CD4+ T-cells and a risk of falling into the category of immunological non-responders [106]. Concurrently, the genetic diversity of the anellome in patients on ART remains elevated compared to healthy individuals [62], indicating a persistent, albeit improved, ability of anelloviruses to evade the immune response even under conditions of HIV virological suppression.

Interpreting the clinical significance of the anellome requires contextual consideration. The apparent paradox identified in the cohort of pediatric long-term non-progressors, where enrichment with anelloviruses was associated with a favorable outcome in contrast to the classical negative correlation in adults which highlights the complexity and ambiguity of their biological role [108]. This contrast suggests that anelloviruses may be not merely a passive marker but an active participant in immune interactions, whose ultimate effect depends on a combination of host factors such as age, immune system status, and the composition of the individual anellome, represented by strains with varying immunomodulatory properties (e.g., differing CpG island composition) [59].

Furthermore, even within adult cohorts, the strength and predictive value of the correlation between anellovirus load (particularly TTV) and immune status can vary. As noted in the review of ART response, seemingly conflicting findings exist—for instance, on whether baseline TTV load predicts CD4+ T-cell recovery [72,106]. These discrepancies underscore that the anellome’s role as a biomarker is modulated by clinical and methodological variables. Key sources of inconsistency include the heterogeneity of study populations (e.g., differences in the definition of immunological non-response, timing of ART initiation, and co-infections) and a lack of standardization in virome quantification methods (e.g., variability in qPCR assays targeting different genomic regions) [113,114,116]. Therefore, a precise interpretation of anellome data must account for this clinical and technical context.

Despite these contextual nuances, the accumulated data provide compelling evidence supporting the potential clinical utility of the anellome as an integrated biomarker of immune competence in HIV infection. Therefore, based on the correlative evidence, anellovirus load/diversity is a candidate tool for future clinical management. This is underscored by the established association between high baseline TTV load and poorer CD4+ T-cell recovery [106], as well as the dynamics of anellovirus suppression during ART [72]. Its envisioned roles in risk stratification, therapeutic decision-making, and non-invasive monitoring (e.g., via saliva) [115] await confirmation in studies that first establish robust, standardized measurement methods and validate their predictive clinical value.

In this context, translating anellome research to resource-limited settings will require the development of affordable, simple diagnostic platforms. Promising candidates for such decentralized, point-of-care anellovirus testing include loop-mediated isothermal amplification (LAMP) and microchip electrophoresis (ME). LAMP amplifies target DNA at a single temperature without a thermocycler and can be coupled with visual readouts (e.g., a color change), making it suitable for use by non-specialists [121]. ME allows for portable, low-cost nucleic acid separation [122]. While primarily qualitative, these methods are promising methods for semi quantitative measurement of anellovirus DNA (for instance, LAMP’s time-to-positive threshold may correlate broadly with viral titer). However, their application for anellovirus monitoring would need to overcome specific challenges: the semi quantitative nature yields approximate viral load estimates, and interpretation must account for confounding patient factors, such as smoking or alcohol use, which are known to independently modulate anellovirus titers and immune status.

The optimal breadth of anellovirus detection for immune monitoring remains an open question. Recent data suggest that the clinical utility of anelloviruses as immune biomarkers may be enhanced by assays that detect not only TTV but also TTMV and possibly TTMDV. In a cohort study, combined monitoring of these three genera increased overall anellovirus detection to ~96%, capturing immune status information missed when testing for TTV alone [123]. Notably, TTMV dynamics often mirrored those of TTV, suggesting it could serve as an alternative marker in the absence of detectable TTV, whereas TTMDV appeared less consistent. These observations raise the possibility that multi-genus panels (e.g., TTV and TTMV) might improve the coverage and robustness of anellome-based monitoring. For HIV infection, this implies that a broader virome assessment could yield a more inclusive biomarker, especially for individuals whose virome is not dominated by *Alphatorquevirus*.

Future research should focus on standardizing methods for anellome measurement and conducting in-depth studies of the immunomodulatory interactions between specific anellovirus genotypes and the immune system of HIV-infected individuals, considering their diverse evasion mechanisms [1] and immunomodulatory properties [59].

## 6. Conclusions

Analysis of the literature indicates that viruses of the family *Anelloviridae* are a key component of the human virome, closely integrated into immune homeostasis. During HIV infection, anelloviruses, particularly those of the genus *Alphatorquevirus* (TTV), exhibit a strong association with the functional state of the immune system. Their viral load and taxonomic diversity rapidly increase in the early stages of HIV infection, correlating with HIV viremia and a decline in CD4+ T-cell count, which indicates a weakening of immune control.

Therefore, anelloviruses, particularly TTV, have emerged as candidate biomarkers for assessing functional immune competence in people living with HIV. The cumulative data provide a strong rationale for investigating anellome monitoring, which could offer prognostic insights into antiretroviral therapy efficacy and the risk of incomplete immunological recovery. However, translating this potential into clinical practice requires overcoming significant hurdles, including the standardization of measurement techniques, validation in large prospective cohorts, and the definition of clinically actionable thresholds. Success in these areas could pave the way for more personalized management approaches in the future.

## 7. Future Directions

While current data provide a strong proof of concept, translating anellovirus research into clinical practice of monitoring the course of HIV infection is a long-term endeavor. The immediate focus must be on methodical, targeted studies to develop standardized tools and generate the evidence necessary for future clinical integration.

The translation of anellome research into clinical practice requires a clear, multi-stage roadmap, beginning with methodological standardization. First, standardization of methods for the quantitative and qualitative analysis of the anellome is required. This includes the development of unified PCR assays for the routine quantification of TTV viral load, as well as the creation of comprehensive sequencing pipelines to capture the diversity of the *Anelloviridae* and track individual anellovirus lineages (e.g., those with pro- or anti-inflammatory CpG motifs). This standardization would enable the establishment of clear TTV viral load thresholds associated with the risk of immunological non-response to ART. Second, standardized methods are a critical prerequisite for resolving the current uncertainty regarding potential geographical and population-based variations in anellome baseline characteristics, which must be understood before these biomarkers can be applied globally in HIV management.

Another crucial fundamental question involves the potential interaction between HIV, APOBEC3, and anelloviruses. This represents a notable knowledge gap. Specifically, it remains unknown whether and how HIV infection (and Vif function) modulates APOBEC3 activity directed against anellovirus genomes. To address this, future longitudinal cohort studies could adopt an integrated approach, combining: (I) host genotyping for relevant APOBEC3 variants (e.g., the APOBEC3B deletion); (II) sequencing of anellovirus genomes with analysis for APOBEC-signature mutations; and (III) collection of concurrent HIV clinical data (viral load, CD4/CD8 counts). Such multifaceted analyses would help clarify molecular link between Vif-mediated suppression of APOBEC3 and anellome dynamics.

Secondly, a key objective is to conduct longitudinal studies to validate the predictive role of the anellome. It is necessary to determine whether baseline TTV levels could inform decisions for earlier treatment initiation or the selection of a more intensive ART regimen in patients showing signs of profound immune exhaustion. Furthermore, expanded research involving different ART regimens may be warranted to explore the potential for using anellome data to guide ART regimen and dosage adjustments.

Thirdly, defining the most informative anellovirus profile. Building on this, a critical research direction is to systematically compare the predictive value of different anellovirus genera—individually and in combination. Future studies should assess whether incorporating TTMV measurement, particularly in individuals with low or absent TTV viremia, adds prognostic value for key clinical endpoints in HIV, such as the timing of ART initiation, the risk of immunological non-response, or disease progression.

Lastly, fundamental research should be focused on deciphering the functional interplay of HIV and anellovirus co-infection. A crucial step is investigating how TTV modulator proteins influence the course of HIV infection. Particular attention must be given to the paradox wherein anelloviruses suppress the NF-κB signaling pathway, which is involved in HIV replication, yet the family *Anelloviridae* is associated with HIV disease progression. This is especially evident in contrast to pegiviruses (*Pegivirus* genus, *Flaviviridae* family), which are associated with a better prognosis in HIV infection. Understanding the molecular interactions between *Anelloviridae* and HIV could open avenues for utilizing anelloviruses not merely as biomarkers but also as potential therapeutic targets to modulate HIV infection in conjunction with ART.

## Data Availability

No new data were created or analyzed in this study. Data sharing is not applicable to this article.

## Abbreviations

The following abbreviations are used in this manuscript:

AIDS	Acquired Immunodeficiency Syndrome
ART	Antiretroviral Therapy
GALT	Gut-Associated Lymphoid Tissue
HIV	Human Immune Deficiency Virus
LTR	Long Terminal Repeat
ORF	Open Reading Frame
PAMP	Pathogen-Associated Molecular Pattern
PBMCs	Peripheral Blood Mononuclear Cells
PRRs	Pattern Recognition Receptors
SIV	Simian Immunodeficiency Vir
ssDNA	Single-Stranded Circular DNA
TAIP	TTV-Derived Apoptosis-Inducing Protein
TLR	Toll-Like Receptor
TTMDV	Torque Teno Midi Virus
TTMV	Torque Teno Mini Virus
TTV	Torque Teno Virus
UTR	Untranslated Region

## References

[1] Liou S., Boggavarapu R., Cohen N.R., Zhang Y., Sharma I., Zeheb L., Mukund Acharekar N., Rodgers H.D., Islam S., Pitts J. (2024). Structure of Anellovirus-like Particles Reveal a Mechanism for Immune Evasion. Nat. Commun..

[2] Modha S., Hughes J., Orton R.J., Lytras S. (2025). Expanding the Genomic Diversity of Human Anelloviruses. Virus Evol..

[3] Laubscher F., Kaiser L., Cordey S. (2024). SCANellome V2: Update of the Primate Anellovirus Reference Sequences Database. Viruses.

[4] Sabbaghian M., Gheitasi H., Shekarchi A.A., Tavakoli A., Poortahmasebi V. (2024). The Mysterious Anelloviruses: Investigating Its Role in Human Diseases. BMC Microbiol..

[5] Mueller B., Maerz A., Doberstein K., Finsterbusch T., Mankertz A. (2008). Gene Expression of the Human Torque Teno Virus Isolate P/1C1. Virology.

[6] Varsani A., Opriessnig T., Celer V., Maggi F., Okamoto H., Blomström A.-L., Cadar D., Harrach B., Biagini P., Kraberger S. (2021). Taxonomic Update for Mammalian Anelloviruses (Family Anelloviridae). Arch. Virol..

[7] Phumiphanjarphak W., Parkbhorn J., Ngamphiw C., Tongsima S., Aiewsakun P. (2025). Discovery of Diverse Anellovirus Sequences in Thai Human Sequencing Data. Microbiol. Spectr..

[8] Do E.D., Holland S.C., Kaelin E.A., Mitchell C., Soria J., La Rosa A., Ticona E., Coombs R.W., Frenkel L.M., Bull M.E. (2024). Genome Sequences of Human Anelloviruses in the *Lamedtorquevirus*, *Memtorquevirus*, and *Samektorquevirus* Genera Identified from the Female Genital Tract. Microbiol. Resour. Announc..

[9] Kaczorowska J., Deijs M., Klein M., Bakker M., Jebbink M.F., Sparreboom M., Kinsella C.M., Timmerman A.L., Van Der Hoek L. (2022). Diversity and Long-Term Dynamics of Human Blood Anelloviruses. J. Virol..

[10] da Costa M.R., da Costa I.P., Devalle S., de Castro A.R.C.M., Freitas S.Z. (2012). Prevalence and Genetic Diversity of Torque Teno Virus in Patients with Systemic Lupus Erythematosus in a Reference Service in Mato Grosso Do Sul. Rev. Bras. Reumatol..

[11] Gergelyjr P., Pullmann R., Stancato C., Otvosjr L., Koncz A., Blazsek A., Poor G., Brown K., Phillips P., Perl A. (2005). Increased Prevalence of Transfusion-Transmitted Virus and Cross-Reactivity with Immunodominant Epitopes of the HRES-1/P28 Endogenous Retroviral Autoantigen in Patients with Systemic Lupus Erythematosus. Clin. Immunol..

[12] Maggi F., Andreoli E., Riente L., Meschi S., Rocchi J., Delle Sedie A., Vatteroni M.L., Ceccherini-Nelli L., Specter S., Bendinelli M. (2007). Torquetenovirus in Patients with Arthritis. Rheumatology.

[13] Timmerman A.L., Schönert A.L.M., van der Hoek L. (2024). Anelloviruses versus Human Immunity: How Do We Control These Viruses?. FEMS Microbiol. Rev..

[14] Okoye A.A., Picker L.J. (2013). CD 4^+^ T-cell Depletion in HIV Infection: Mechanisms of Immunological Failure. Immunol. Rev..

[15] Li G., Lou Y., Ma J., Cheng L., Yu H., Tsahouridis O., He X., Funaki M., Ahodantin J., Bi W. (2025). Depletion of Plasmacytoid Dendritic Cells Rescues HIV-Reactive Stem-like CD8^+^ T Cells during Chronic HIV-1 Infection. Sci. Transl. Med..

[16] Lin X., Song B., Cao L., Zhang L., Liu S., Wang X., Chen X., Li S. (2025). PD-1 Suppression Enhances HIV Reactivation and T-Cell Immunity via MAPK/NF-κB Signaling. Eur. J. Med. Res..

[17] Hu W., Li Y.-J., Zhen C., Wang Y.-Y., Huang H.-H., Zou J., Zheng Y.-Q., Huang G.-C., Meng S.-R., Jin J.-H. (2022). CCL5-Secreting Virtual Memory CD8+ T Cells Inversely Associate with Viral Reservoir Size in HIV-1−Infected Individuals on Antiretroviral Therapy. Front. Immunol..

[18] Ma J., Wang G., Zhu X., Li L., Wang L., Hao L., Gao L., Ma W., Zhang N. (2023). Combining CD4 Count, CD8 Count and CD4/CD8 Ratio to Predict Risk of Mortality among HIV-Positive Adults after Therapy: A Group-Based Multi-Trajectory Analysis. Front. Immunol..

[19] Ron R., Martínez-Sanz J., Herrera S., Ramos-Ruperto L., Díez-Vidal A., Sainz T., Álvarez-Díaz N., Correa-Pérez A., Muriel A., López-Alcalde J. (2024). CD4/CD8 Ratio and CD8+ T-Cell Count as Prognostic Markers for Non-AIDS Mortality in People Living with HIV. A Systematic Review and Meta-Analysis. Front. Immunol..

[20] Yan L., Xu K., Xiao Q., Tuo L., Luo T., Wang S., Yang R., Zhang F., Yang X. (2023). Cellular and Molecular Insights into Incomplete Immune Recovery in HIV/AIDS Patients. Front. Immunol..

[21] Timmerman A.L., Commandeur L., Deijs M., Burggraaff M.G.J.M., Lavell A.H.A., Van Der Straten K., Tejjani K., Van Rijswijk J., Van Gils M.J., Sikkens J.J. (2024). The Impact of First-Time SARS-CoV-2 Infection on Human Anelloviruses. Viruses.

[22] Zheng H., Ye L., Fang X., Li B., Wang Y., Xiang X., Kong L., Wang W., Zeng Y., Ye L. (2007). Torque Teno Virus (SANBAN Isolate) ORF2 Protein Suppresses NF-κB Pathways via Interaction with IκB Kinases. J. Virol..

[23] Arze C.A., Springer S., Dudas G., Patel S., Bhattacharyya A., Swaminathan H., Brugnara C., Delagrave S., Ong T., Kahvejian A. (2021). Global Genome Analysis Reveals a Vast and Dynamic Anellovirus Landscape within the Human Virome. Cell Host Microbe.

[24] Kapoor A., Kumar A., Simmonds P., Bhuva N., Singh Chauhan L., Lee B., Sall A.A., Jin Z., Morse S.S., Shaz B. (2015). Virome Analysis of Transfusion Recipients Reveals a Novel Human Virus That Shares Genomic Features with Hepaciviruses and Pegiviruses. mBio.

[25] Kosulin K., Kernbichler S., Pichler H., Lawitschka A., Geyeregger R., Witt V., Lion T. (2018). Post-Transplant Replication of Torque Teno Virus in Granulocytes. Front. Microbiol..

[26] Okamoto H., Takahashi M., Nishizawa T., Tawara A., Sugai Y., Sai T., Tanaka T., Tsuda F. (2000). Replicative Forms of TT Virus DNA in Bone Marrow Cells. Biochem. Biophys. Res. Commun..

[27] Li Y., Cao L., Ye M., Xu R., Chen X., Ma Y., Tian R.-R., Liu F.-L., Zhang P., Kuang Y.-Q. (2022). Plasma Virome Reveals Blooms and Transmission of Anellovirus in Intravenous Drug Users with HIV-1, HCV, and/or HBV Infections. Microbiol. Spectr..

[28] Inami T., Konomi N., Arakawa Y., Abe K. (2000). High Prevalence of TT Virus DNA in Human Saliva and Semen. J. Clin. Microbiol..

[29] Fornai C., Maggi F., Vatteroni M.L., Pistello M., Bendinelli M. (2001). High Prevalence of TT Virus (TTV) and TTV-Like Minivirus in Cervical Swabs. J. Clin. Microbiol..

[30] Brani P., Manzoor H.Z., Spezia P.G., Vigezzi A., Ietto G., Dalla Gasperina D., Minosse C., Bosi A., Giaroni C., Carcano G. (2025). Torque Teno Virus: Lights and Shades. Viruses.

[31] Mariscal L.F., López-Alcorocho J.M., Rodríguez-Iñigo E., Ortiz-Movilla N., De Lucas S., Bartolomé J., Carreño V. (2002). TT Virus Replicates in Stimulated but Not in Nonstimulated Peripheral Blood Mononuclear Cells. Virology.

[32] Zhou J.Z., Way S.S., Chen K. (2018). Immunology of the Uterine and Vaginal Mucosae. Trends Immunol..

[33] Gonzalez S.M., Aguilar-Jimenez W., Su R.-C., Rugeles M.T. (2019). Mucosa: Key Interactions Determining Sexual Transmission of the HIV Infection. Front. Immunol..

[34] Matsubara H. (2001). Existence of TT Virus DNA and TTV-like Mini Virus DNA in Infant Cord Blood: Mother-to-Neonatal Transmission. Hepatol. Res..

[35] Bagaglio S., Sitia G., Prati D., Cella D., Hasson H., Novati R., Lazzarin A., Morsica G. (2002). Mother-to-Child Transmission of TT Virus: Sequence Analysis of Non-Coding Region of TT Virus in Infected Mother-Infant Pairs. Arch. Virol..

[36] Gerner P., Oettinger R., Gerner W., Falbrede J., Wirth S. (2000). Mother-to-Infant Transmission of TT Virus: Prevalence, Extent and Mechanism of Vertical Transmission. Pediatr. Infect. Dis. J..

[37] Kyathanahalli C., Snedden M., Hirsch E. (2021). Human Anelloviruses: Prevalence and Clinical Significance During Pregnancy. Front. Virol..

[38] Wylie K.M., Wylie T.N., Cahill A.G., Macones G.A., Tuuli M.G., Stout M.J. (2018). The Vaginal Eukaryotic DNA Virome and Preterm Birth. Am. J. Obstet. Gynecol..

[39] McCann A., Ryan F.J., Stockdale S.R., Dalmasso M., Blake T., Ryan C.A., Stanton C., Mills S., Ross P.R., Hill C. (2018). Viromes of One Year Old Infants Reveal the Impact of Birth Mode on Microbiome Diversity. PeerJ.

[40] Kaczorowska J., Cicilionytė A., Timmerman A.L., Deijs M., Jebbink M.F., Van Goudoever J.B., Van Keulen B.J., Bakker M., Van Der Hoek L. (2022). Early-Life Colonization by Anelloviruses in Infants. Viruses.

[41] Kaczorowska J., Cicilionytė A., Wahdaty A.F., Deijs M., Jebbink M.F., Bakker M., Van Der Hoek L. (2022). Transmission of Anelloviruses to HIV-1 Infected Children. Front. Microbiol..

[42] Beller L., Deboutte W., Vieira-Silva S., Falony G., Tito R.Y., Rymenans L., Yinda C.K., Vanmechelen B., Van Espen L., Jansen D. (2022). The Virota and Its Transkingdom Interactions in the Healthy Infant Gut. Proc. Natl. Acad. Sci. USA.

[43] Zaki M., El-Sabbagh A., Salam M., Mefreh M. (2021). Molecular Study of Torque Teno Virus in Patients with Acute Gastroenteritis. Clin. Lab..

[44] Maggi F., Pifferi M., Fornai C., Andreoli E., Tempestini E., Vatteroni M., Presciuttini S., Marchi S., Pietrobelli A., Boner A. (2003). TT Virus in the Nasal Secretions of Children with Acute Respiratory Diseases: Relations to Viremia and Disease Severity. J. Virol..

[45] Deng X., Terunuma H., Handema R., Sakamoto M., Kitamura T., Ito M., Akahane Y. (2000). Higher Prevalence and Viral Load of TT Virus in Saliva than in the Corresponding Serum: Another Possible Transmission Route and Replication Site of TT Virus. J. Med. Virol..

[46] Nokhova A.R., Dubovitskiy N.A., Derko A.A., Khozyainova A.A., Kurskaya O.G., Shestopalov A.M., Sharshov K.A. (2025). Genetic Characteristics of Anelloviruses Detected in Individual Viromes of Children with Acute Respiratory Symptoms Using the Metagenomic Approach. Acta Virol..

[47] Cao L., Ma Y., Wan Z., Li B., Tian W., Zhang C., Li Y. (2023). Longitudinal Anellome Dynamics in the Upper Respiratory Tract of Children with Acute Respiratory Tract Infections. Virus Evol..

[48] Chikasue K., Kimura M., Ikeda K., Ohnishi T., Kawanishi S., Iio T., Kataoka M., Arao Y. (2012). Detection of Torque Teno Virus DNA in Exhaled Breath by Polymerase Chain Reaction. Acta Med. Okayama..

[49] Kaczorowska J., van der Hoek L. (2020). Human Anelloviruses: Diverse, Omnipresent and Commensal Members of the Virome. FEMS Microbiol. Rev..

[50] Dodi G., Attanasi M., Di Filippo P., Di Pillo S., Chiarelli F. (2021). Virome in the Lungs: The Role of Anelloviruses in Childhood Respiratory Diseases. Microorganisms.

[51] Reyes N.S., Spezia P.G., Jara R., Filippini F., Boccia N., García G., Hermida E., Poletta F.A., Pistello M., Laham G. (2024). Torque Teno Virus (TTV) in Renal Transplant Recipients: Species Diversity and Variability. Viruses.

[52] Castain L., Petrier M., Bulteau S., Peltier C., Poulain C., Bouras M., Imbert-Marcille B.-M., Poschmann J., Roquilly A., Bressollette-Bodin C. (2024). Association of Dynamics of Anellovirus Loads With Hospital-Acquired Pneumonia in Patients With Brain Injury During the Intensive Care Unit Stay. J. Infect. Dis..

[53] Doorenbos C.S.E., Jonker J., Hao J., Gore E.J., Kremer D., Knobbe T.J., De Joode A.A.E., Sanders J.S.F., Thaunat O., Niesters H.G.M. (2023). Smoking, Alcohol Intake and Torque Teno Virus in Stable Kidney Transplant Recipients. Viruses.

[54] Gauthier T. (2015). Prenatal Alcohol Exposure and the Developing Immune System. Alcohol Res. Curr. Rev..

[55] Saint-André V., Charbit B., Biton A., Rouilly V., Possémé C., Bertrand A., Rotival M., Bergstedt J., Patin E., Albert M.L. (2024). Smoking Changes Adaptive Immunity with Persistent Effects. Nature.

[56] Tyschik E.A., Rasskazova A.S., Degtyareva A.V., Rebrikov D.V., Sukhikh G.T. (2018). Torque Teno Virus Dynamics during the First Year of Life. Virol. J..

[57] Cebriá-Mendoza M., Beamud B., Andreu-Moreno I., Arbona C., Larrea L., Díaz W., Sanjuán R., Cuevas J.M. (2023). Human Anelloviruses: Influence of Demographic Factors, Recombination, and Worldwide Diversity. Microbiol. Spectr..

[58] Martelli F., Macera L., Spezia P.G., Medici C., Pistello M., Guasti D., Romagnoli P., Maggi F., Giannecchini S. (2018). Torquetenovirus Detection in Exosomes Enriched Vesicles Circulating in Human Plasma Samples. Virol. J..

[59] Freer G., Maggi F., Pifferi M., Di Cicco M.E., Peroni D.G., Pistello M. (2018). The Virome and Its Major Component, Anellovirus, a Convoluted System Molding Human Immune Defenses and Possibly Affecting the Development of Asthma and Respiratory Diseases in Childhood. Front. Microbiol..

[60] Ball J.K., Curran R., Berridge S., Grabowska A.M., Jameson C.L., Thomson B.J., Irving W.L., Sharp P.M. (1999). TT Virus Sequence Heterogeneity in Vivo: Evidence for Co-Infection with Multiple Genetic Types. J. Gen. Virol..

[61] Timmerman A.L., Kaczorowska J., Deijs M., Bakker M., Van Der Hoek L. (2022). Control of Human Anelloviruses by Cytosine to Uracil Genome Editing. mSphere.

[62] Kaczorowska J., Timmerman A.L., Deijs M., Kinsella C.M., Bakker M., Van Der Hoek L. (2023). Anellovirus Evolution during Long-Term Chronic Infection. Virus Evol..

[63] Kandathil A.J., Clipman S.J., Anantharam R., Duchen D., Cox A.L., Larman H.B., Thomas D.L. (2025). Antibody-Mediated Control of Anellovirus Infection: Evidence from People Who Inject Drugs. J. Virol..

[64] Venkataraman T., Swaminathan H., Arze C.A., Jacobo S.M., Bhattacharyya A., David T., Nawandar D.M., Delagrave S., Mani V., Yozwiak N.L. (2022). Comprehensive Profiling of Antibody Responses to the Human Anellome Using Programmable Phage Display. Cell Rep..

[65] Kakkola L., Bondén H., Hedman L., Kivi N., Moisala S., Julin J., Ylä-Liedenpohja J., Miettinen S., Kantola K., Hedman K. (2008). Expression of All Six Human Torque Teno Virus (TTV) Proteins in Bacteria and in Insect Cells, and Analysis of Their IgG Responses. Virology.

[66] Gunderson S., Eskew A.M., Stoutenburg D., Riley J.K., Stout M.J., Schrimpf J., Jungheim E.S., Wylie K.M. (2022). Association of the Human Semen DNA Virome with Successful in Vitro Fertilization. FS Sci..

[67] Maggi F., Fornai C., Vatteroni M.L., Siciliano G., Menichetti F., Tascini C., Specter S., Pistello M., Bendinelli M. (2001). Low Prevalence of TT Virus in the Cerebrospinal Fluid of Viremic Patients with Central Nervous System Disorders. J. Med. Virol..

[68] Rocchi J., Ricci V., Albani M., Lanini L., Andreoli E., Macera L., Pistello M., Ceccherini-Nelli L., Bendinelli M., Maggi F. (2009). Torquetenovirus DNA Drives Proinflammatory Cytokines Production and Secretion by Immune Cells via Toll-like Receptor 9. Virology.

[69] Pifferi M., Maggi F., Cristofano C.D., Cangiotti A.M., Nelli L.C., Bevilacqua G., Macchia P., Bendinelli M., Boner A.L. (2008). Torquetenovirus Infection and Ciliary Dysmotility in Children with Recurrent Pneumonia. Pediatr. Infect. Dis. J..

[70] Pifferi M., Maggi F., Caramella D., De Marco E., Andreoli E., Meschi S., Macchia P., Bendinelli M., Boner A.L. (2006). High Torquetenovirus Loads Are Correlated With Bronchiectasis and Peripheral Airflow Limitation in Children. Pediatr. Infect. Dis. J..

[71] Takahashi M., Asabe S., Gotanda Y., Kishimoto J., Tsuda F., Okamoto H. (2002). TT Virus Is Distributed in Various Leukocyte Subpopulations at Distinct Levels, with the Highest Viral Load in Granulocytes. Biochem. Biophys. Res. Commun..

[72] Abbate I., Rozera G., Cimini E., Carletti F., Tartaglia E., Rubino M., Pittalis S., Esvan R., Gagliardini R., Mondi A. (2023). Kinetics of TTV Loads in Peripheral Blood Mononuclear Cells of Early Treated Acute HIV Infections. Viruses.

[73] Desai M., Pal R., Deshmukh R., Banker D. (2005). Replication of TT Virus in Hepatocyte and Leucocyte Cell Lines. J. Med. Virol..

[74] Tang J.Y., Chen T.B., Kouznetsova V.L., Tsigelny I.F. (2025). Anelloviruses and Cancer. J. Infect. Dis..

[75] Nawandar D.M., Trivedi M., Bounoutas G., Lebo K., Prince C., Scano C., Agarwal N., Ozturk E., Yu J., Arze C.A. (2022). Human Anelloviruses Produced by Recombinant Expression of Synthetic Genomes. bioRxiv.

[76] De Smit M.H., Noteborn M.H.M., De Villiers E.-M., Hausen H.Z. (2009). Apoptosis-Inducing Proteins in Chicken Anemia Virus and TT Virus. TT Viruses.

[77] Swinkels H.M., Nguyen A.D., Gulick P.G. (2025). HIV and AIDS. StatPearls.

[78] Calado M., Pires D., Conceição C., Ferreira R., Santos-Costa Q., Anes E., Azevedo-Pereira J.M. (2023). Cell-to-Cell Transmission of HIV-1 and HIV-2 from Infected Macrophages and Dendritic Cells to CD4+ T Lymphocytes. Viruses.

[79] Kulkarni R., Prasad A. (2017). Exosomes Derived from HIV-1 Infected DCs Mediate Viral Trans-Infection via Fibronectin and Galectin-3. Sci. Rep..

[80] Sharp P.M., Hahn B.H. (2011). Origins of HIV and the AIDS Pandemic. Cold Spring Harb. Perspect. Med..

[81] Campbell-Yesufu O.T., Gandhi R.T. (2011). Update on Human Immunodeficiency Virus (HIV)-2 Infection. Clin. Infect. Dis..

[82] Vidya Vijayan K.K., Karthigeyan K.P., Tripathi S.P., Hanna L.E. (2017). Pathophysiology of CD4+ T-Cell Depletion in HIV-1 and HIV-2 Infections. Front. Immunol..

[83] Deeks S.G., Tracy R., Douek D.C. (2013). Systemic Effects of Inflammation on Health during Chronic HIV Infection. Immunity.

[84] Mu W., Patankar V., Kitchen S., Zhen A. (2024). Examining Chronic Inflammation, Immune Metabolism, and T Cell Dysfunction in HIV Infection. Viruses.

[85] Zhang Q.-S., Wang J.-N., Yang T.-L., Li S.-Y., Li J.-Q., Liu D.-N., Shang H., Zhang Z.-N. (2025). SHMT2 Regulates CD8+ T Cell Senescence via the Reactive Oxygen Species Axis in HIV-1 Infected Patients on Antiretroviral Therapy. eBioMedicine.

[86] Masur H., Brooks J.T., Benson C.A., Holmes K.K., Pau A.K., Kaplan J.E. (2014). Prevention and Treatment of Opportunistic Infections in HIV-Infected Adults and Adolescents: Updated Guidelines From the Centers for Disease Control and Prevention, National Institutes of Health, and HIV Medicine Association of the Infectious Diseases Society of America. Clin. Infect. Dis..

[87] Yarchoan R., Uldrick T.S. (2018). HIV-Associated Cancers and Related Diseases. N. Engl. J. Med..

[88] Kemnic T.R., Gulick P.G. (2025). HIV Antiretroviral Therapy. StatPearls.

[89] Aquaro S., Borrajo A., Pellegrino M., Svicher V. (2020). Mechanisms Underlying of Antiretroviral Drugs in Different Cellular Reservoirs with a Focus on Macrophages. Virulence.

[90] Ma Y., Zhang M., Wang Z., Cao L., Li Y., Wan Z., Kane Y., Wang G., Li X., Zhang C. (2025). Short-Term Antiretroviral Therapy May Not Correct the Dysregulations of Plasma Virome and Cytokines Induced by HIV-1 Infection. Virulence.

[91] Bhagchandani T., Ul Haque M.M., Sharma S., Malik M.Z., Ray A.K., Kaur U.S., Rai A., Verma A., Sawlani K.K., Chaturvedi R. (2024). Plasma Virome of HIV-Infected Subjects on Suppressive Antiretroviral Therapy Reveals Association of Differentially Abundant Viruses with Distinct T-Cell Phenotypes and Inflammation. Curr. Genom..

[92] Vegas Rodriguez A., Velez De Mendizábal N., Girish S., Trocóniz I.F., Feigelman J.S. (2025). Modeling the Interplay Between Viral and Immune Dynamics in HIV: A Review and Mrgsolve Implementation and Exploration. Clin. Transl. Sci..

[93] Kazer S.W., Walker B.D., Shalek A.K. (2020). Evolution and Diversity of Immune Responses during Acute HIV Infection. Immunity.

[94] Murzin A.I., Elfimov K.A., Gashnikova N.M. (2024). The Proviral Reservoirs of Human Immunodeficiency Virus (HIV) Infection. Pathogens.

[95] Rocafort M., Noguera-Julian M., Rivera J., Pastor L., Guillén Y., Langhorst J., Parera M., Mandomando I., Carrillo J., Urrea V. (2019). Evolution of the Gut Microbiome Following Acute HIV-1 Infection. Microbiome.

[96] Lapa D., Del Porto P., Minosse C., D’Offizi G., Antinori A., Capobianchi M.R., Visco-Comandini U., McPhee F., Garbuglia A.R., Zaccarelli M. (2021). Clinical Relevance of Torque Teno Virus (TTV) in HIV/HCV Coinfected and HCV Monoinfected Patients Treated with Direct-Acting Antiviral Therapy. J. Clin. Med..

[97] Shibayama T., Masuda G., Ajisawa A., Takahashi M., Nishizawa T., Tsuda F., Okamoto H. (2001). Inverse Relationship between the Titre of TT Virus DNA and the CD4 Cell Count in Patients Infected with HIV. AIDS.

[98] Tarancon-Diez L., Carrasco I., Montes L., Falces-Romero I., Vazquez-Alejo E., Jiménez De Ory S., Dapena M., Iribarren J.A., Díez C., Ramos-Ruperto L. (2024). Torque Teno Virus: A Potential Marker of Immune Reconstitution in Youths with Vertically Acquired HIV. Sci. Rep..

[99] Esser P.L., Quintanares G.H.R., Langhans B., Heger E., Böhm M., Jensen B.-E.O.L.E., Esser S., Lübke N., Fätkenheuer G., Lengauer T. (2024). Torque Teno Virus Load Is Associated with Centers for Disease Control and Prevention Stage and CD4+ Cell Count in People Living with Human Immunodeficiency Virus but Seems Unrelated to AIDS-Defining Events and Human Pegivirus Load. J. Infect. Dis..

[100] Devalle S., Niel C. (2004). Distribution of TT Virus Genomic Groups 1–5 in Brazilian Blood Donors, HBV Carriers, and HIV-1-infected Patients. J. Med. Virol..

[101] Elesinnla A.R., Adeleye I.A., Ayolabi C.I., Bessong P.O. (2020). Prevalence of Torque Viruses in HIV-Infected and Non-HIV-Infected Nigerian Subjects: Analysis of near-Full-Length Genome Sequences. Arch. Virol..

[102] Liu K., Li Y., Xu R., Zhang Y., Zheng C., Wan Z., Li H., Yang Z., Jin X., Hao P. (2021). HIV-1 Infection Alters the Viral Composition of Plasma in Men Who Have Sex with Men. mSphere.

[103] Boukadida C., Peralta-Prado A., Chávez-Torres M., Romero-Mora K., Rincon-Rubio A., Ávila-Ríos S., Garrido-Rodríguez D., Reyes-Terán G., Pinto-Cardoso S. (2024). Alterations of the Gut Microbiome in HIV Infection Highlight Human Anelloviruses as Potential Predictors of Immune Recovery. Microbiome.

[104] Kaelin E.A., Mitchell C., Soria J., La Rosa A., Ticona E., Coombs R.W., Frenkel L.M., Bull M.E., Lim E.S. (2025). Longitudinal Cervicovaginal Bacteriome and Virome Alterations Associate with Discordant Shedding and ART Duration in Women Living with HIV in Peru. Nat. Commun..

[105] Fogli M., Torti C., Malacarne F., Fiorentini S., Albani M., Izzo I., Giagulli C., Maggi F., Carosi G., Caruso A. (2012). Emergence of Exhausted B Cells in Asymptomatic HIV-1-Infected Patients Naïve for HAART Is Related to Reduced Immune Surveillance. Clin. Dev. Immunol..

[106] Schmidt L., Jensen B.-E.O., Walker A., Keitel-Anselmino V., Di Cristanziano V., Böhm M., Knops E., Heger E., Kaiser R., De Luca A. (2021). Torque Teno Virus Plasma Level as Novel Biomarker of Retained Immunocompetence in HIV-Infected Patients. Infection.

[107] Guedes M.C.S., Lopes-Araujo H.F., Dos Santos K.F., Simões E., Carvalho-Silva W.H.V., Guimarães R.L. (2025). How to Properly Define Immunological Nonresponse to Antiretroviral Therapy in People Living with HIV? An Integrative Review. Front. Immunol..

[108] Mwesigwa S., Williams L., Retshabile G., Katagirya E., Mboowa G., Mlotshwa B., Kyobe S., Kateete D.P., Wampande E.M., Wayengera M. (2021). Unmapped Exome Reads Implicate a Role for Anelloviridae in Childhood HIV-1 Long-Term Non-Progression. Npj Genom. Med..

[109] Ji J., Guo C., Li Z., Cai M., Wang R., Chen X., Zhang Y., Wu H., Zhang T., Zhang Y. (2025). Rapid Initiation of Antiretroviral Therapy Suppresses T Cell Pathological Proliferation and Improves Immune Recovery in People Living with HIV. Infect. Drug Resist..

[110] Aprilia A., Handono K., Sujuti H., Sabarudin A., Winaris N. (2024). sCD163, sCD28, sCD80, and sCTLA-4 as Soluble Marker Candidates for Detecting Immunosenescence. Immun. Ageing.

[111] Semnani-Azad Z., Blanco Mejia S., Connelly P.W., Bazinet R.P., Retnakaran R., Jenkins D.J.A., Harris S.B., Hanley A.J. (2021). The Association of Soluble CD163, a Novel Biomarker of Macrophage Activation, with Type 2 Diabetes Mellitus and Its Underlying Physiological Disorders: A Systematic Review. Obes. Rev..

[112] Durda P., Raffield L.M., Lange E.M., Olson N.C., Jenny N.S., Cushman M., Deichgraeber P., Grarup N., Jonsson A., Hansen T. (2022). Circulating Soluble CD163, Associations With Cardiovascular Outcomes and Mortality, and Identification of Genetic Variants in Older Individuals: The Cardiovascular Health Study. J. Am. Heart Assoc..

[113] Pinto-Cardoso S., Chávez-Torres M., López-Filloy M., Ávila-Ríos S., Romero-Mora K., Peralta-Prado A. (2025). Patterns of Immune Recovery in People Living with HIV Who Initiated Antiretroviral Therapy as Late Presenters. BMC Infect. Dis..

[114] Zhao H., Feng A., Luo D., Yuan T., Lin Y.-F., Ling X., Zhong H., Li J., Li L., Zou H. (2024). Factors Associated with Immunological Non-Response after ART Initiation: A Retrospective Observational Cohort Study. BMC Infect. Dis..

[115] Honorato L., Witkin S.S., Mendes-Correa M.C., Conde Toscano A.L.C., Linhares I.M., De Paula A.V., Paião H.G.O., De Paula V.S., Lopes A.D.O., Lima S.H. (2022). The Torque Teno Virus Titer in Saliva Reflects the Level of Circulating CD4+ T Lymphocytes and HIV in Individuals Undergoing Antiretroviral Maintenance Therapy. Front. Med..

[116] Spezia P.G., Carletti F., Novazzi F., Specchiarello E., Genoni A., Drago Ferrante F., Minosse C., Matusali G., Mancini N., Focosi D. (2024). Torquetenovirus Viremia Quantification Using Real-Time PCR Developed on a Fully Automated, Random-Access Platform. Viruses.

[117] Li L., Deng X., Da Costa A.C., Bruhn R., Deeks S.G., Delwart E. (2015). Virome Analysis of Antiretroviral-Treated HIV Patients Shows No Correlation between T-Cell Activation and Anelloviruses Levels. J. Clin. Virol..

[118] Li J.Z., Segal F.P., Bosch R.J., Lalama C.M., Roberts-Toler C., Delagreverie H., Getz R., Garcia-Broncano P., Kinslow J., Tressler R. (2020). Antiretroviral Therapy Reduces T-Cell Activation and Immune Exhaustion Markers in Human Immunodeficiency Virus Controllers. Clin. Infect. Dis..

[119] Bone B., Cotugno N., Pighi C., Rotili A., Hong S., Carrere L., Morrocchi E., Pascucci G.R., Gao C., Colantoni N. (2025). Distinct Viral Reservoirs and Immune Signatures in Individuals on Long-Term Antiretroviral Therapy with Perinatally Acquired HIV-1. Cell Rep. Med..

[120] Goila-Gaur R., Strebel K. (2008). HIV-1 Vif, APOBEC, and Intrinsic Immunity. Retrovirology.

[121] Matl M., Kellner M.J., Ansah F., Grishkovskaya I., Handler D., Heinen R., Bauer B., Menéndez-Arias L., Auer T.O., Prieto-Godino L.L. (2025). A Lyophilized Open-Source RT-LAMP Assay for Molecular Diagnostics in Resource-Limited Settings. Life Sci. Alliance.

[122] Zeid A.M., Abdussalam A., Hanif S., Anjum S., Lou B., Xu G. (2023). Recent Advances in Microchip Electrophoresis for Analysis of Pathogenic Bacteria and Viruses. Electrophoresis.

[123] Cinti L., Spezia P.G., Roberto P., Russo G., Lai Q., Carillo C., Frasca F., Antonelli G., Maggi F. (2025). Assessment of Torquetenominivirus (TTMV) and Torquetenomidivirus (TTMDV) as Complementary Biomarkers to Torquetenovirus (TTV). Int. J. Mol. Sci..

